# In Situ Converting Conformal Sacrificial Layer Into Robust Interphase Stabilizes Fluorinated Polyanionic Cathodes for Aqueous Sodium‐Ion Storage

**DOI:** 10.1002/advs.202501362

**Published:** 2025-05-02

**Authors:** Peng Gong, Shibo Chai, Xingjie Li, Yibo Dong, Shengjun Zhai, Xihao Chen, Ning Wang, Yuanyuan Li, Jinping Liu

**Affiliations:** ^1^ School of Integrated Circuits Huazhong University of Science and Technology Wuhan 430074 P. R. China; ^2^ School of Chemistry Chemical Engineering and Life Sciences and State Key Laboratory of Advanced Technology for Materials Synthesis and Processing Wuhan University of Technology Wuhan 430070 P. R. China; ^3^ School of Materials Science and Engineering Chongqing University of Arts and Sciences Chongqing 402160 P. R. China; ^4^ School of Science Key Laboratory of High Performance Scientific Computation Xihua University Chengdu 610039 P. R. China

**Keywords:** aqueous sodium‐ion storage, cathode electrolyte interphase, hybrid capacitor, in situ interfacial conversion, sodium vanadium oxy‐fluorophosphates

## Abstract

Sodium vanadium oxy‐fluorophosphates (NVOPF), as typical fluorinated polyanionic compounds, are considered high‐voltage and high‐capacity cathode materials for aqueous sodium‐ion storage. However, the poor cycle life caused by interfacial degradation (especially the attack of specific HF by‐products) greatly hampers their application in aqueous electrolytes. Here, it is shown that in situ converting harmful HF derivate to F‐containing cathode electrolyte interphase (CEI) can overcome the above challenge. As a proof‐of‐concept, a conformal Al_2_O_3_ sacrificial layer is precoated on NVOPF for on‐site generating robust AlF_3_‐rich CEI while eliminating continuous HF release. The evolved CEI chemistry mitigates interfacial side reactions, inhibits vanadium dissolution, and promotes Na^+^ transport kinetics, thus significantly boosting cycling stability (capacity retention rate increased to 3.15 times), rate capability, and even low‐temperature performance (≈1.5 times capacity improvement at −20 °C). When integrated with pseudocapacitive zeolite‐templated carbon anode and adhesive hydrogel electrolyte, a unique 2.3 V quasi‐solid‐state sodium‐ion hybrid capacitor is developed, exhibiting remarkable cycle life (77.0% after 1000 cycles), high energy and power densities, and exceptional safety against extreme conditions. Furthermore, a photovoltaic energy storage module is demonstrated, highlighting the potential use in future smart/microgrids. The work paves new avenues for enabling the use of unstable electrode materials via interfacial engineering.

## Graphical abstract

A unique interfacial conversion concept is proposed to establish robust cathode electrolyte interphase for stabilizing fluorinated polyanionic cathodes in aqueous electrolytes. In a typical case, the conformal Al_2_O_3_ sacrificial layer is predeposited on high‐voltage sodium vanadium oxy‐fluorophosphates cathode, which can progressively react with trace HF derivate to in situ form dense AlF_3_‐rich interphase during initial cycling, leading to significantly improved sodium‐ion storage capability.

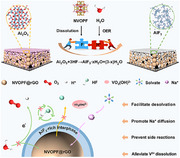

## 1 Introduction

To effectively utilize intermittent renewable energy sources such as wind and solar power, the development of inexpensive grid scale energy storage systems with high energy and power densities has been accelerated.^[^ ^1^ ^]^ Aqueous energy storage devices are promising for such stationary storage applications due to their high safety, environmental friendliness, low cost, and high power density. Particularly, aqueous sodium‐ion storage techniques have gained extensive attention in terms of highly abundant sodium resources.^[^ ^2^ ^]^ In practice, however, it is extremely difficult to achieve competitive energy density for aqueous sodium‐ion storage devices; one key reason is the lack of high potential, high capacity, and highly stable cathode materials.^[^ ^3^ ^]^

Sodium vanadium oxy‐fluorophosphates (Na_3_V_2_O_2x_(PO_4_)_2_F_3−2x_, NVOPF, 0 < x ≤ 1), known as typical fluorinated polyanionic materials, have drawn considerable attention as cathode materials for nonaqueous sodium‐ion batteries owing to their high potential plateaus (the highest approaching 1.2 V versus saturated calomel electrode, SCE), large theoretical specific capacity (≈128 mAh g^−1^), and 3D intrinsic ion transport channels.^[^ ^4^ ^]^ Nonetheless, the extremely poor cycle life has long hampered their use for aqueous sodium‐ion storage. Apart from the common issues of vanadium dissolution induced by active water erosion and oxygen evolution reaction (OER) for vanadium‐based cathodes,^[^ ^5^ ^]^ the attack of trace HF corrosion should also be noted for inherent F‐containing polyanionic materials such as NVOPF. During operation in aqueous electrolytes, spontaneous thermodynamic and electrochemical dissolution inevitably generates hard Lewis‐base F^−^ at first, which will capture H^+^ mainly originating from undesirable OER to form the reactive species of HF acid. Then the generated HF will cause structural degradation via exacerbating vanadium dissolution, leading to the sharp capacity fade.^[^ ^6^ ^]^ Up to now, only several strategies have been proposed to address this tricky issue. For example, a highly concentrated “water‐in‐salt” electrolyte (WISE) with limited active water content was utilized to suppress dissolution,^[^ ^7^ ^]^ organic additives were added to the electrolyte to reduce water activity through reconstruction of H‐bonds network,^[^ ^8^ ^]^ and guest ions were introduced into the V^4+^ sites to enhance crystal intrinsic stability.^[^ ^9^ ^]^ However, the effect was ultimately found to be insufficient; the longest cycle life remains limited to only 100 cycles with a residual capacity of 32 mAh g^−1^. In most cases, in contrast to the general water erosion, the fatal effect of possible HF attack did not arouse sufficient concern. To effectively boost the operation stability of NVOPF in aqueous electrolyte, building a robust, chemically and electrochemically stable, protective interfacial layer on NVOPF is of extreme importance.

Unfortunately, the most adopted surface precoating technique, which typically involves the deposition of materials such as oxides, phosphates, and carbonates,^[^ ^10^ ^]^ has not been demonstrated effective in mitigating the impact of on‐site generated HF. Recently, in situ constructing cathode electrolyte interphase (CEI) has proven to be effective in enhancing the durability of nonaqueous batteries by preventing interfacial reactions;^[^ ^11^ ^]^ F‐containing species (such as NaF) are well‐accepted as one of the key components in the CEI, which possess high chemical/electrochemical stability and low ion diffusion barrier.^[^ ^12^ ^]^ However, such CEI is preferentially originated from the oxidative decomposition of giant anions and/or organic solvents that are generally contained in nonaqueous electrolytes;^[^ ^13^ ^]^ in a common aqueous electrolyte with simple salts (anions), it is inconvenient to in situ form NaF‐rich CEI on cathode by a similar way, especially considering the relatively high solubility of NaF in water. Given that trace F^−^ release is unavoidable during NVOPF cycling in aqueous electrolytes, we wonder whether the released F^−^ can be in situ converted into some kind of insoluble F‐rich interphase that is electronically insulating but ionically conductive. This is believed to kill two birds with one stone, that is, not only on‐site anchor F^−^ to avoid the continuous formation of destructive HF, but also help to prevent the vanadium dissolution from water erosion and OER at the cathode side.

**Scheme 1:**
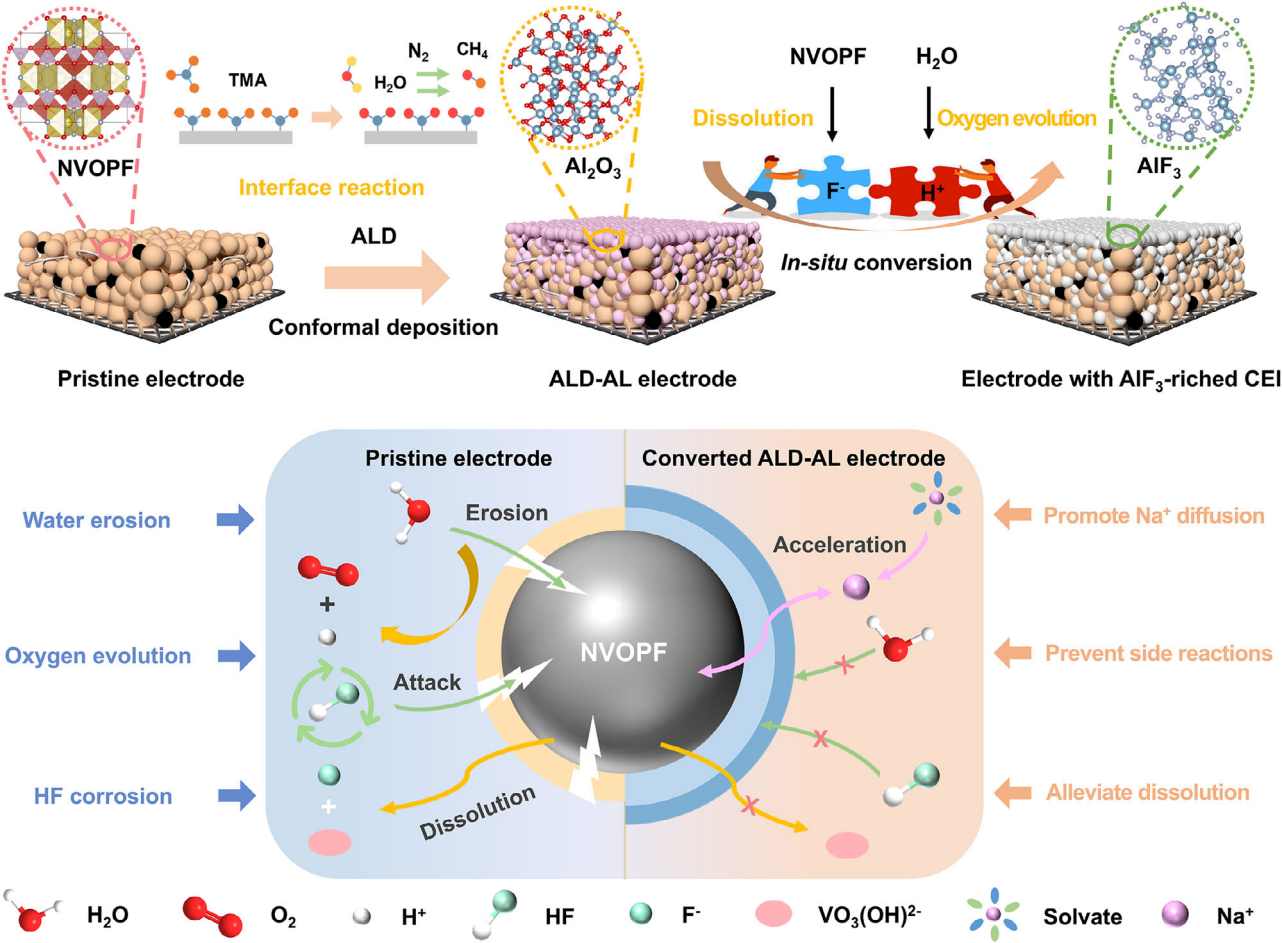
Schematic illustration of the in situ conversion process from ALD‐AL to electrode with AlF_3_‐rich CEI, as well as the merits of the in situ converted electrode.

Herein, we report on an in situ interfacial conversion concept to establish robust CEI for stabilizing F‐containing cathode, which is achieved by pre‐depositing a uniform sacrificial layer on the cathode and further converting it into F‐rich interphase via on‐site immobilizing the released F^−^ from cathode material. In a typical case, we conformally coat common Al_2_O_3_ nano‐layer on a fluorinated polyanionic cathode of NVOPF by atomic layer deposition (ALD) technique (the resulting cathode is denoted as ALD‐AL), which can progressively react with trace HF by‐product to in situ form unusual AlF_3_‐rich interphase during initial cycling process (**Scheme** **1**). The chemically stable interphase isolates the direct contact between NVOPF particles and aqueous electrolytes, thereby mitigating interfacial side reactions and inhibiting vanadium dissolution. Besides, the derivative NaAlO_2_ in AlF_3_‐rich CEI with high ionic conductivity across the interface facilitates Na^+^ diffusion. As a result, the ALD‐AL cathode exhibits much‐improved cycling stability (capacity retention rate after 100 cycles increased to 315%) with a high cut‐off potential of 1.2 V versus SCE; the discharge capacity, rate capability, and even low‐temperature performance (at −20 °C) are also enhanced.

To further demonstrate the superiority of our interfacial conversion strategy, an aqueous sodium‐ion hybrid capacitor (ASIHC), which is anticipated to combine the complementary features of aqueous sodium‐ion batteries and supercapacitors,^[^ ^14^ ^]^ is constructed by pairing ALD‐AL cathode with a pseudocapacitive zeolite‐templated carbon (ZTC) anode. The resulting ASIHC can operate at a high voltage window of 2.3 V, showing a boosted capacity retention of 71.2% after 500 cycles, prominently outperforming the device using a pristine cathode (59.9% after only 100 cycles). The established quasi‐solid‐state ASIHC pouch cell also exhibits long cycling stability (77.0% after 1000 cycles), high energy and power densities, as well as exceptional flexibility and safety, which has been readily integrated with photovoltaic packs and demonstrated practical renewable energy storage.

## 2 Results and Discussion

### 2.1 Fabrication and Characterization of ALD‐AL Cathode

NVOPF material was synthesized via interfacial redox reaction between trivalent vanadium (V^3+^) precursor and graphene oxide (GO) (see details in Experimental Section), and the specific chemical component was determined as Na_3_V_2_O_2_(PO_4_)_2_F;^[^ ^15^ ^]^ the reduced graphene oxide (rGO) network in situ formed during the synthesis is believed to enhance the electronic conductivity of NVOPF. The resulting NVOPF@rGO composite demonstrates a sphere shape with diameters of several hundred nanometers (Figure S1, Supporting Information), with all the X‐ray diffraction (XRD) peaks well indexed to the standard NVOPF (Figure S2, Supporting Information; JCPDS Card No. 89–8485). Al_2_O_3_ layer was directly coated on the as‐prepared NVOPF@rGO cathode using ALD at 150 °C (Figure S3, Supporting Information). Note that the interparticle electronic pathways within the ALD‐AL cathode are already formed prior to the ALD deposition, which in principle will not be affected during the subsequent conformal and penetrating coating of Al_2_O_3_ onto NVOPF@rGO particles.

**Figure 1:**
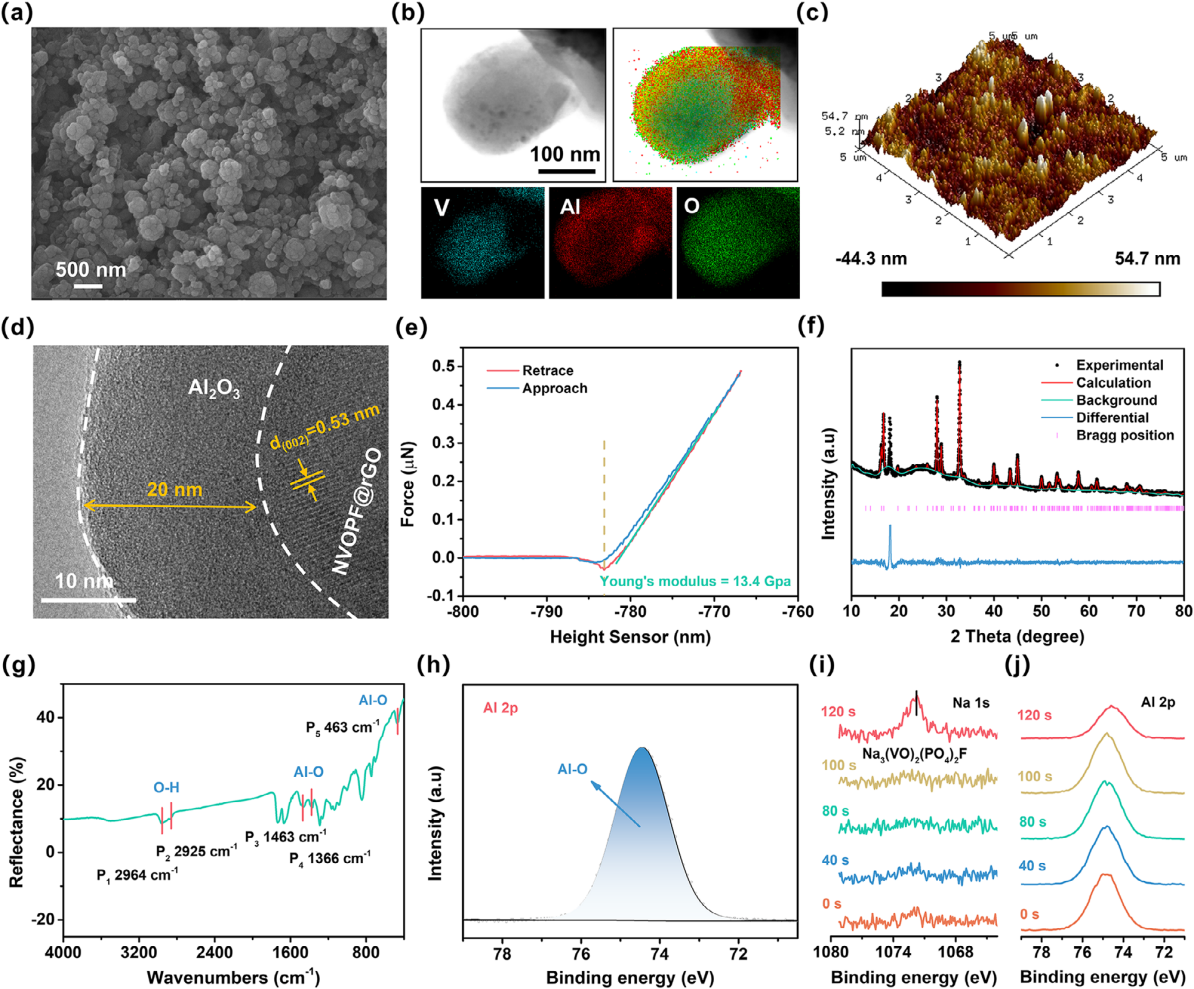
a) SEM image of ALD‐AL cathode. b) EDS elemental mapping of individual NVOPF@rGO nanospheres. c) AFM image, d) TEM image, and e) Force‐separation curve of ALD‐AL cathode. f) XRD pattern with Rietveld refinement, g) ATR‐FTIR, and h) Al 2p XPS spectra of ALD‐AL cathode. XPS depth spectra of i) Na 1s and j) Al 2p collected from ALD‐AL cathode.

Scanning electron microscopy (SEM) was employed to observe the morphology. The ALD‐AL and pristine electrodes show no visible difference in terms of surface morphology (**Figure** **1**a; Figure S4, Supporting Information). To determine the existence of Al_2_O_3_ layer, elemental mapping was performed by energy dispersive X‐ray spectrometer (EDS). As illustrated in Figure 1b, Al and O elements are homogeneously distributed on NVOPF@rGO particle surface. The atomic force microscopy (AFM) analysis further verifies the similar roughness of the two samples (Figure 1c; Figure S5, Supporting Information). These provide clear evidence for successfully deposited Al_2_O_3_ on the electrode surface with uniform and conformal characteristics. Typically, the thickness of Al_2_O_3_ layer after 150 cycles of ALD deposition is measured to be ≈20 nm (Figure 1d), slightly thinner than the value (21 nm) calculated based on the deposition rate of the ALD process. Young's modulus was measured to evaluate the mechanical property by AFM in the PeakForce QNM mode. As shown in Figure 1e, the average Young's modulus of Al_2_O_3_ layer is 13.4 GPa, which is generally conducive to relieving volume change and preventing harmful species permeation.^[^ ^16^ ^]^

The phases and compositions of ALD‐AL cathode were investigated by XRD, no peaks associated with Al_2_O_3_ are observed (Figure S6, Supporting Information), indicating that the deposited Al_2_O_3_ is probably amorphous. Then the crystal structures of pristine and ALD‐AL cathodes were identified by Rietveld refinement (Figure 1f; Figure S7, Supporting Information). The corresponding crystallographic parameters are displayed in Table S1 (Supporting Information), and the low *R*‐factor values verify the reliability of the structural analyses.^[^ ^17^ ^]^ It is noteworthy that the lattice parameters of ALD‐AL electrode (*a* = 9.044 Å, *c* = 10.653 Å) are almost the same to the pristine electrode (*a* = 9.045 Å, *c* = 10.654 Å), demonstrating the negligible effect of ALD on the NVOPF crystal structure. Note that the additional peak observed at 18.2° is attributed to polytetrafluoroethylene (PTFE) binder (JCPDS Card No. 47–2217). Attenuated Total Reflection Fourier Transform Infrared (ATR‐FTIR) was conducted to analyze the surface bonding states of the ALD‐AL cathode, the peaks at 463, 1366, and 1463 cm^−1^ are characteristic peaks of Al─O bond in Al_2_O_3_ (Figure 1g). X‐ray photoelectron spectroscopy (XPS) analysis also confirms the finding (Figure S8, Supporting Information); the high‐resolution Al 2p spectrum can be decomposed into one prominent peak at 74.6 eV corresponding to Al─O bond (Figure 1h), which is also verified by the deconvoluted peak at 531.1 eV in the O 1s spectrum (Figure S9, Supporting Information).

Furthermore, the penetrating distribution of Al_2_O_3_ in ALD‐AL cathode was confirmed by XPS depth profile analysis (etching rate: 0.2 nm s^−1^; Figure 1i). The Na 1s signal of NVOPF is not detected during the initial etching period of 0—100 s (≈20 nm depth) but arises after 120 s etching (≈24 nm depth), implying that there is indeed Al_2_O_3_ layer depositing on the electrode surface with a thickness of ≈20 nm, consistent with the above TEM observation. The intensity of Al_2_O_3_ characteristic peak remains consistent upon etching of 0—100 s (Figure 1j), indicating a uniform distribution in the depth direction; then it shows a decrease instead of disappearance after 120 s etching, suggesting a deeper diffusion of Al_2_O_3_ even into the inner electrode. This penetration is also supported by cross‐sectional SEM images and the corresponding EDS mapping results in Figure S10 (Supporting Information), and is believed to improve the cycling stability of NVOPF to the utmost extent.

### 2.2 Electrochemical Performance of ALD‐AL Cathode

**Figure 2:**
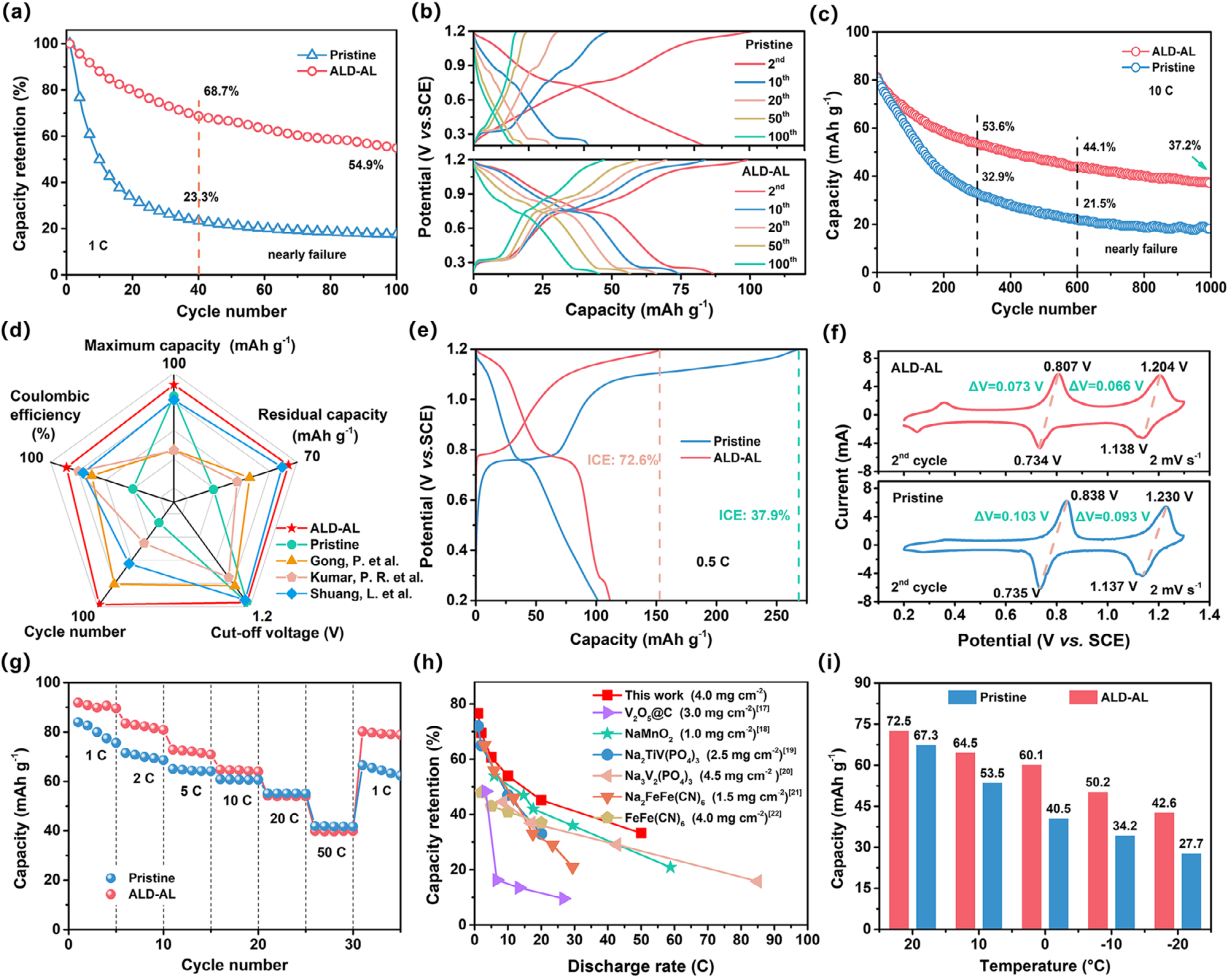
a) Cycle stability comparison of pristine and ALD‐AL cathodes. b) Corresponding GCD profiles after different cycles. c) Long‐term cycling performance comparison. d) Electrochemical performance comparison of ALD‐AL cathode with previous reports. e) The first‐cycle GCD curves, f) CV curves, and g) rate capability comparison of pristine and ALD‐AL cathodes. h) Rate capability comparison of ALD‐AL cathode with other typical cathodes. i) Capacity comparison of pristine and ALD‐AL cathodes at different temperatures.

The effect of Al_2_O_3_ layer on the electrochemical behaviors of ALD‐AL cathode was studied by comparing with the pristine cathode. It was found that the ALD‐AL cathode with 150 cycles of ALD deposition demonstrated superior comprehensive performance in terms of cycling stability and coulombic efficiency (Figure S11, Supporting Information); thus, this optimal cathode was used for the next comparisons. As displayed in **Figure** **2**a, the pristine electrode exhibits rapid capacity fade (≈23.3% after only 40 cycles) at 1 C (1 C = 128 mA g^−1^); by contrast, ALD‐AL electrode demonstrates significantly improved cycling stability (≈54.9% after 100 cycles) within an ultrawide potential window of 0.2–1.2 V versus SCE. Figure 2b shows the evolution of galvanostatic charge–discharge (GCD) curves during cycling, ALD‐AL electrode also shows quite a small voltage fade, while the electrode voltage plateau disappears very fast without ALD coating. Even at a high rate of 10 C (Figure 2c), ALD‐AL electrode exhibits better long‐term durability as compared to pristine electrodes (44.1% vs 21.5%, after 600 cycles). Significantly, compared with the previous reports on stabilizing NVOPF in aqueous electrolytes (Figure 2d and Table S2, Supporting Information),^[^ ^7^, ^8^, ^18^ ^]^ our ALD‐AL cathode demonstrates superior comprehensive performance. Obviously, these improvements should be attributed to the homogeneous Al_2_O_3_ coating layer, and the underlying mechanism will be elaborated in the following section.

The first‐cycle GCD curves tested at 0.5 C are shown in Figure 2e. At this low rate, the initial coulombic efficiency (ICE) of ALD‐AL cathode is 72.6%, obviously surpassing the 37.9% recorded for the pristine cathode, indicating significantly suppressed interfacial side reactions (Figure S12, Supporting Information). The ICE of ALD‐AL cathode fails to reach a higher value, probably due to the formation of Na‐containing interphases during the charging process which will be discussed later. And thanks to the improved interfacial wettability (Figure S13, Supporting Information), ALD‐AL cathode delivers an initial discharge capacity of 113.6 mAh g^−1^, which is 11.8% greater than the pristine electrode (101.6 mAh g^−1^). It is worth noting that the capacity contribution of Al_2_O_3_ is negligible (Figure S14, Supporting Information). Figure 2f illustrates the cyclic voltammetry (CV) curves obtained at a scan rate of 2 mV s^−1^ within the voltage range of 0.2–1.3 V (vs SCE). The ALD‐AL cathode exhibits similar CV profiles to the pristine cathode, with two dominant pairs of redox peaks (≈1.1/1.2 V and ≈0.7/0.8 V) corresponding to the two‐step reversible sodiation/desodiation reactions occurring at Na1 site and Na2 site in NVOPF, respectively.^[^ ^7^ ^]^ Nevertheless, it possesses a smaller electrochemical polarization, which should benefit from the aforementioned improved interfacial wettability and the estimated higher Na^+^ diffusion coefficient of ≈1.01 × 10^−10^ cm^2^ s^−1^ (Figures S15 and S16, Supporting Information). The small redox peaks at 0.23/0.33 V should originate from reversible Na^+^ insertion/extraction in the trace VO_2_ derivate.^[^ ^18^ ^]^ The rate performances of the two electrodes ranging from 1 to 50 C are further presented in Figure 2g, and the corresponding GCD profiles are given in Figure S17 (Supporting Information). In general, the ALD‐AL cathode demonstrates better rate capability than the pristine one; especially at rates of 1—10 C, it delivers much higher capacities. Figure 2h compares ALD‐AL electrode with some typical reported Na‐ion storage cathodes in aqueous electrolytes, such as transition metal oxide of V_2_O_5_@C^[^ ^19^ ^]^ and NaMnO_2_,^[^ ^20^ ^]^ polyanion compounds of Na_2_TiV(PO_4_)_3_^[^ ^21^ ^]^ and Na_3_V_2_(PO_4_)_3_,^[^ ^22^ ^]^ and Prussian blue analogs of Na_2_FeFe(CN)_6_^[^ ^23^ ^]^ and FeFe(CN)_6_.^[^ ^24^ ^]^ The data clearly evidence that our ALD‐AL cathode with a relatively high mass loading of ≈4 mg cm^−2^ exhibits the highest rate capability (highest capacity retention with increasing the rate), which will benefit the kinetics match with capacitive anode for constructing advanced ASIHC. More interestingly, with surface coating our ALD‐AL cathode demonstrates better low‐temperature performance (Figure 2i). Upon decreasing the temperature from 20 to −20 °C, the pristine cathode delivers significantly reduced capacities, with a value as low as 27.3 mAh g^−1^ at −20 °C; by contrast, ALD‐AL cathode provides a much higher capacity of 42.6 mAh g^−1^ at −20 °C. In addition, our ALD‐AL cathode shows a much smaller voltage polarization increase in GCD profiles with the decrease of temperature to −20 °C (Figure S18, Supporting Information). The above results unambiguously indicate the importance of interface engineering via specific conformal coating for NVOPF cathode operating in aqueous electrolytes.

### 2.3 The Evolution of Al_2_O_3_ Interphase Chemistry

**Figure 3:**
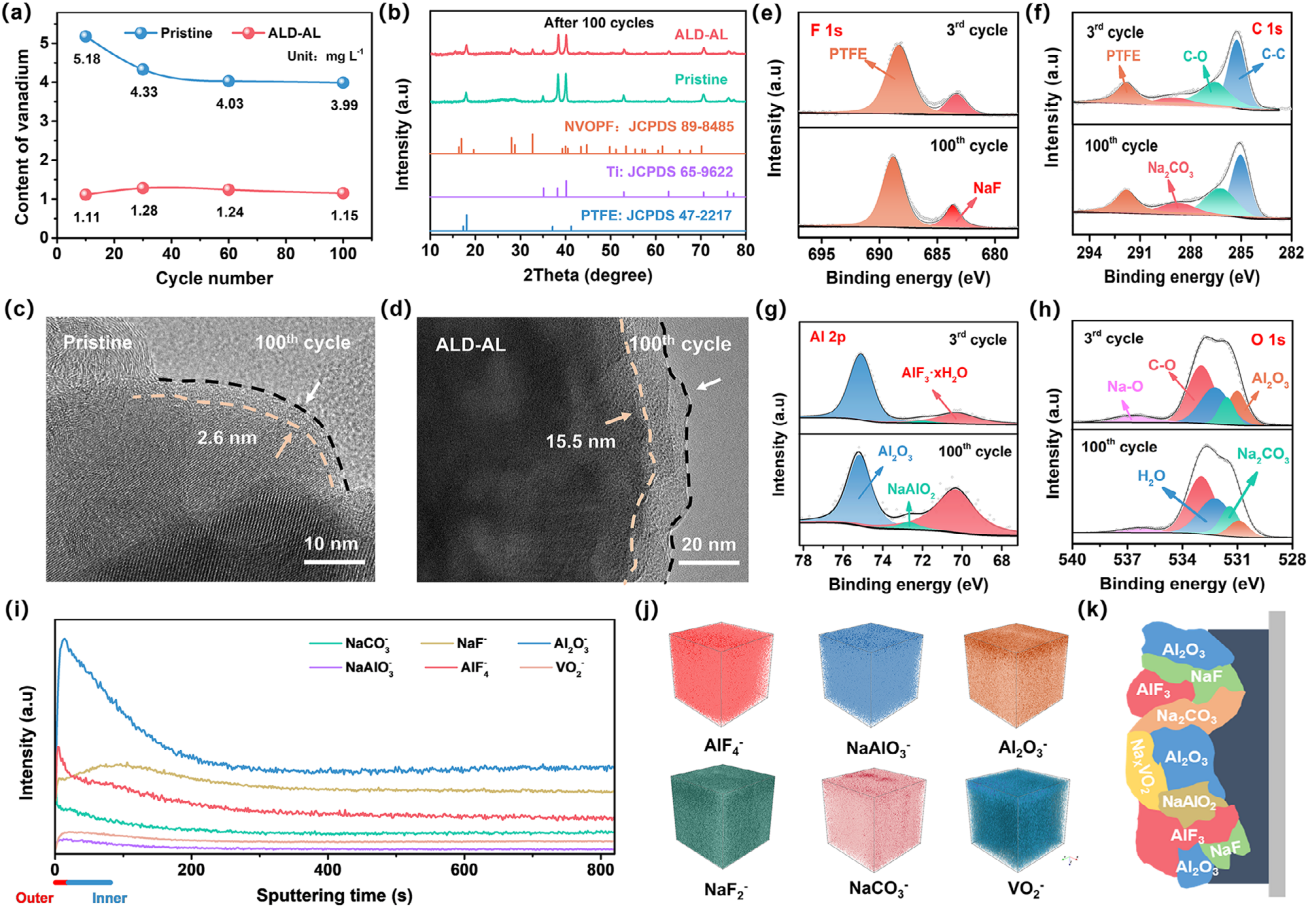
a) Dissolved vanadium content comparison of pristine and ALD‐AL cathodes after different cycles. b) XRD patterns of pristine and ALD‐AL cathodes after 100 cycles. HRTEM images of c) pristine cathode and d) ALD‐AL cathode after 100 cycles. e) F 1s and f) C 1s XPS spectra of pristine cathode after 3 cycles and 100 cycles. g) Al 2p and h) O 1s XPS spectra of ALD‐AL cathode after 3 cycles and 100 cycles. i) TOF‐SIMS intensity depth profiles, and j) 3D distribution of various species. k) Schematic diagram of the reconstructed CEI.

We found that the pristine cathode (without ALD coating) displays serious surface pulverization after 100 cycles, whereas the ALD‐AL cathode almost maintains the original particle morphology of the fresh electrode (Figure S19, Supporting Information). An inductively coupled plasma optical emission spectrometer (ICP‐OES) was thus conducted to determine the electrolyte composition during cycling, checking the vanadium dissolution degree of cathode materials. As illustrated in **Figure** **3**a, ALD‐AL electrode indeed has much less vanadium dissolution, consistent with the morphology evolution. Likewise, ALD‐AL electrode maintains its structure well while the pristine one almost loses the crystalline feature after 100 cycles, as confirmed by XRD results (Figure 3b). Apparently, the precoated Al_2_O_3_ interphase has helped to inhibit vanadium dissolution into aqueous electrolyte and thus stabilized the cathode structure.

To further understand the underlying mechanism of vanadium dissolution inhibition, interphase evolution was monitored (Figure 3c–k). Figure 3c shows the high‐resolution TEM (HRTEM) image of the cycled pristine cathode, a thin interphase of ≈2.6 nm exists on the electrode surface, mainly composed of Na_2_CO_3_ and NaF (Figure 3e,f). Since Na_2_CO_3_ and NaF are unstable in aqueous electrolytes due to their high solubility (Figure S20, Supporting Information), a pristine cathode should suffer continuous destruction from active H_2_O molecules and acidic species (HF, H_2_CO_3_) during the electrochemical process. By contrast, the ALD‐AL cathode is well covered by a thicker interphase (≈15.5 nm) after 100 cycles (Figure 3d). It is noted that the interphase thickness on the cycled ALD‐AL cathode is smaller than the originally coated Al_2_O_3_, implying possible interface evolution during cycling. Indeed, HF by‐products can also be generated during the initial cycling process of ALD‐AL cathode (Figure S21, Supporting Information), which, in principle, can react with the Al_2_O_3_ coating layer.

Therefore, additional studies were conducted to analyze the chemical components of the surface of the cycled ALD‐AL cathode. In the Al 2p spectra (Figure 3g), except for Al_2_O_3_, the peaks belonging to AlF_3_·*x*H_2_O (70.6 eV) and NaAlO_2_ (72.7 eV) are clearly detected, which are also evidenced by deconvolution of the O 1s and F 1s spectra (Figure 3h; Figure S22, Supporting Information). These XPS results clearly demonstrate the in situ interfacial reactions of the precoated Al_2_O_3_ layer with electrolyte and NVPOF, and the possible reaction processes are elaborated in Figure S23 (Supporting Information). AlF_3_ is well known to have high ionic conductivity and electrochemical stability,^[^ ^25^ ^]^ which is believed to not only facilitate the ion diffusion at the interface but also prevent the cathode material from being attacked by an electrolyte. In addition, a certain amount of Na_2_CO_3_ and NaF is also detected on ALD‐AL cathode surface. These sodium salts acting as sodium ionic conductors will further promote Na^+^ diffusion of the cathode. Since the interphase chemistry can affect the internal microstructure of CEI, the time‐of‐flight secondary ion mass spectroscopy (TOF‐SIMS) was performed to further figure out the configuration of the reconstructed interphase. Figure 3i shows the intensity depth profiles of different secondary ion fragments for 850 s sputtering time (the analyzed area is 60 × 60 µm); the 3D spatial distribution plots are given in Figure 3j. The inorganic sputtered fragments of AlF_4_^−^ (representing AlF_3_) and NaAlO_3_^−^ (representing NaAlO_2_) are formed by the reactions between Al_2_O_3_ and derivates in electrolyte, NaCO_3_^−^ (representing Na_2_CO_3_) and NaF^−^ (representing NaF) should be derived from electrode‐electrolyte interactions, and VO_2_^−^ (representing Na_x_VO_2_) originates from the reduction product of dissolved vanadium ion (VO_3_(OH)_2_^−^).^[^ ^18^ ^]^ It can be seen that the outer CEI layer of ALD‐AL cathode is mainly composed of AlF_4_^−^, NaCO_3_^−^, and VO_2_^−^ species, while the inner layer contains a large amount of NaF^−^ as well as a little NaAlO_3_^−^. The microscopic component and structure schematic of the CEI is illustrated in Figure 3k. Note that species derived from NaClO_4_ electrolytes can also be detected, such as NaCl and AlCl_3_, but their contents are even lower than trace NaAlO_2_ (Figure S24, Supporting Information).

Based on the above investigations, the pre‐coated Al_2_O_3_ on ALD‐AL cathode acts as a unique sacrificial layer, which can be in situ converted into robust AlF_3_‐rich CEI via the interfacial reaction with the released F^−^ and electrolyte. The evolved and reconstructed CEI is envisioned to have potential merits and functions as follows. First, the CEI layer isolates the direct contact between NVOPF particles and electrolytes to weaken water erosion and OER, leading to reduced vanadium dissolution. Second, upon cycles, the conversion from Al_2_O_3_ to AlF_3_/NaAlO_2_ eliminates the released trace harmful HF by‐product, further preventing structural deterioration. Third, the outer AlF_3_ protects ionically conductive Na_2_CO_3_/NaF species in CEI from dissociation by active H_2_O, leading to promoted interfacial Na^+^ transport kinetics.

### 2.4 The Function Mechanisms of AlF_3_‐Rich Interphase

**Figure 4:**
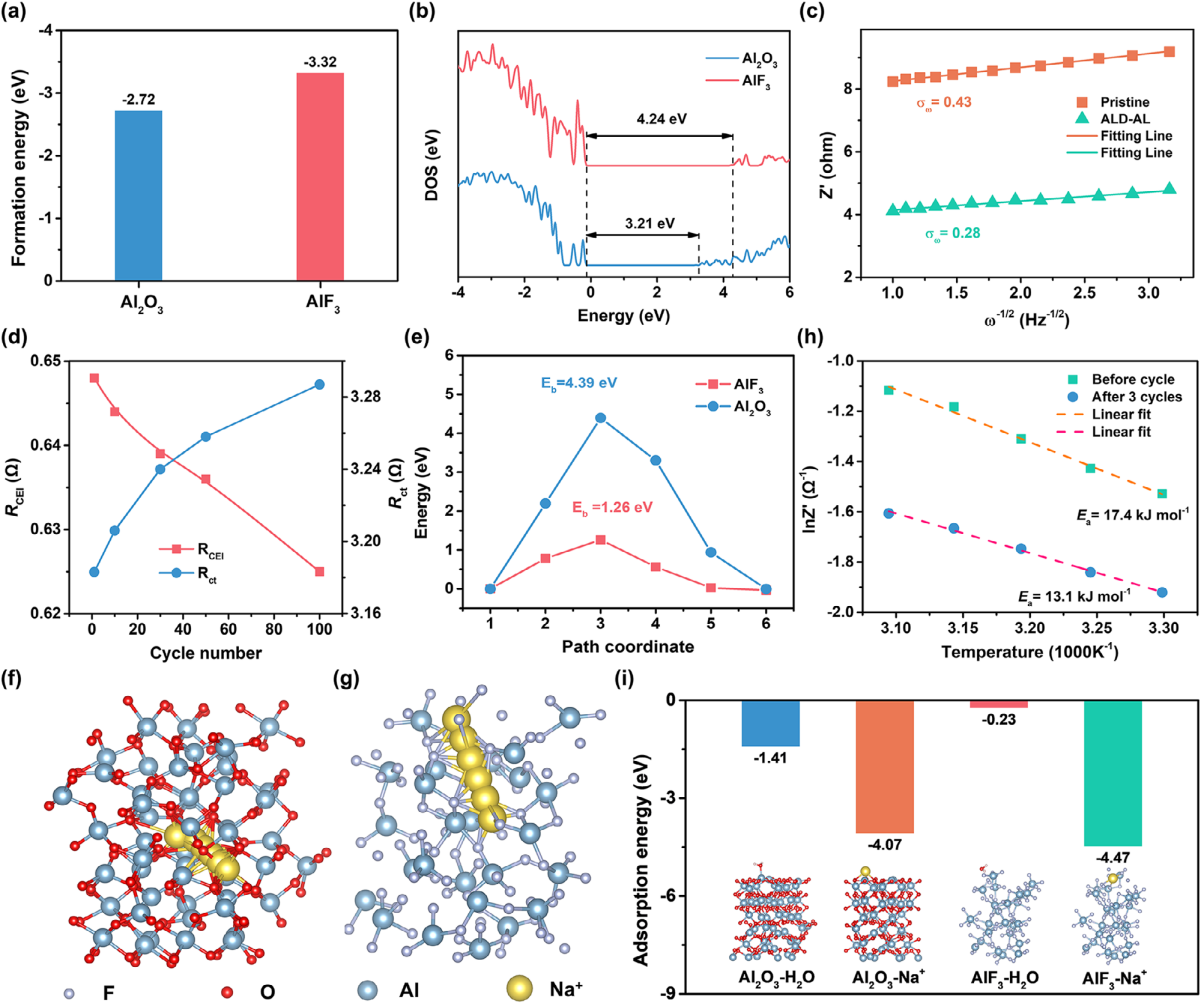
a) The formation energy and b) density of states of Al_2_O_3_ and AlF_3_. c) The relationship between *Z*′ and *ω* ^−1/2^ in low‐frequency region. d) *R*_CEI_ and *R*_ct_ of ALD‐AL cathode after different cycles. e) Na^+^ migration energy, and schematic diagrams of possible Na^+^ migration path in f) Al_2_O_3_ and g) AlF_3_. h) The desolvation activation energies are calculated by the Arrhenius equation. i) Absorption energies of H_2_O and Na^+^ on the surface of Al_2_O_3_ and AlF_3_.

Density functional theory (DFT) calculations were employed to verify the superiority of in situ formed AlF_3_‐rich interphase over the original Al_2_O_3_. It has been accepted that structural stability is a fundamental requirement for ideal CEI, which can be assessed by analyzing the formation energy (*E*_f_). The smaller *E*_f_ means that a mixed system needs to release more energy to the outside when atoms are combined from the elementary substance into a crystal, thus the interatomic action is stronger and the thermodynamic stability is higher. As shown in **Figure** **4**a, AlF_3_ possesses smaller *E*_f_ than Al_2_O_3_ (−3.23 vs −2.72 eV), therefore, the crystal structure of AlF_3_ is more stable. Besides, AlF_3_ is calculated to own a wider bandgap than that of Al_2_O_3_ in the energy band structure, signifying better electronic insulation as CEI (Figure 4b).

To gain deeper insight into the influence of interphase on the interfacial reaction kinetics, electrochemical impedance spectra (EIS) of the cathodes before and after cycling were systematically recorded. In Nyquist plots (Figure S25, Supporting Information), the intercept in *x*‐axis is the internal resistance (*R*_s_) of the electrolyte and electrode; there is a single semicircle related to charge transfer resistance (*R*_ct_) in the region of high to medium frequency, with a straight line representing the Warburg resistance (*Z*_w_) at the low‐frequency region. After three cycles, another semicircle corresponding to interfacial resistance (*R*_CEI_) is observed due to the formation of CEI. The Nyquist plots were fitted with an equivalent circuit model (the inset in Figure S26, Supporting Information), and the fitted values are given in Table S3. Obviously, ALD‐AL cathode exhibits lower *R*_CEI_ and *R*_ct_ than that of the pristine cathode, implying faster ion diffusion and charge transfer. Based on the relationship between real impedance (Z′) and the square root of angular frequency (ω^−1/2^) in Figure 4c, Na^+^ diffusion coefficient (*D*_Na+_) can be readily calculated. The *D*_Na+_ of ALD‐AL electrode reaches 5.76 × 10^−9^ cm^2^ s^−1^ after three cycles, which is more than twice that of the pristine electrode (2.45 × 10^−9^ cm^2^ s^−1^).

The Nyquist plots of ALD‐AL cathode after different cycles (1, 10, 30, 50, 100) were further recorded and fitted (Figure S27 and Table S4, Supporting Information). The *R*_CEI_ value decreases constantly during the cycling process (Figure 4d), which verifies the dynamic evolution from the pre‐coated Al_2_O_3_ layer to AlF_3_‐rich CEI with better Na^+^ diffusion capability.^[^ ^26^ ^]^ The neglectable increase of *R*_ct_ should be due to the slight cathode material structural change that influences the charge transfer process. As illustrated in Figure 4e–g, theoretical calculations also demonstrate that the Na^+^ diffusion barrier in AlF_3_ is much lower than Al_2_O_3_ (1.26 vs 4.39 eV), indicating that AlF_3_ interphase indeed facilitates faster Na^+^ migration. Furthermore, the impact of interphase evolution on Na^+^ desolvation was also evaluated. The activation energy for Na^+^ desolvation (*E*_a_) was calculated according to the classic Arrhenius equation. Based on the *R*_ct_ from EIS recorded at temperatures of 30—50 °C (Figure S28, Supporting Information), *E*_a_ values of ALD‐AL cathode before and after three cycles are estimated as 17.4 and 13.1 kJ mol^−1^ (Figure 4h), respectively, revealing that the reconstructed AlF_3_‐rich interphase possesses lower energy barrier for ion desolvation. The superiority of AlF_3_ layer on Na^+^ desolvation was further confirmed by DFT calculations. As shown in Figure 4i, AlF_3_ manifests stronger adsorption capability toward Na^+^ and weaker adsorption capability toward H_2_O molecules than Al_2_O_3_. The differences in the adsorption energy imply that AlF_3_, as the key component in the interphase, can help to facilitate the desolvation of water molecules from the hydrated Na^+^ at the electrode‐electrolyte interface.

Overall, the function mechanisms of in situ formed AlF_3_‐rich interphase have been well understood. Essentially, the better thermodynamic stability and electric insulation of AlF_3_‐rich CEI can effectively prevent unfavorable interfacial side reactions, enabling better cycling stability and high coulombic efficiency of the ALD‐AL cathode. In addition, the interphase endowed faster interfacial kinetics, beneficial from the much lower interfacial resistance and desolvation energy, should mainly account for the observed larger discharge capacity, and better rate and low‐temperature performances.

### 2.5 Assembly and Performance of ALD‐AL//ZTC Full Cells

Considering the high Na^+^ storage potential and significantly boosted cycling stability and reversible capacity, our ALD‐AL cathode holds great promise in constructing high‐voltage and high‐energy aqueous sodium‐ion storage devices. As an example, we thus assembled an ASIHC by paring our cathode with a pseudocapacitive ZTC anode. The morphology and charge storage performance of ZTC anode were first investigated (Figure S29, Supporting Information). The anode consists of convex polyhedral nanoparticles with sizes of 150–300 nm (Figure S29a, Supporting Information) and elements of C and O (Figure S29b, Supporting Information). There are abundant hydroxyl functional groups on the surface (Figure S29c, Supporting Information), which offer great potential for redox reactions after suitable functionalization treatments. As expected, the CV current is dominated by a pseudocapacitive process (Figure S30, Supporting Information) after further simple activation (Figure S31, Supporting Information), which is also evidenced by the quasi‐rectangular CV with broad redox peaks and quasi‐triangular GCD profiles (Figure S29d,e, Supporting Information). The pseudocapacitance originates from the reversible sodiation/desodiation reaction of polar C═O functional groups.^[^ ^18^ ^]^ Thanks to the excellent structure stability of carbon materials, the activated ZTC anode delivers a highly reversible capability of 107.6 mAh g^−1^ at 0.5 A g^−1^ (≈298.0 F g^−1^) in the potential range of −1.1 to 0.2 V, prominent rate performance (≈70% capacity retained with the current increased 40 times to 20 A g^−1^) (Figure S29f, Supporting Information), and excellent cyclic stability (limited capacity fade after 900 cycles) (Figure S29f, Supporting Information).

**Figure 5:**
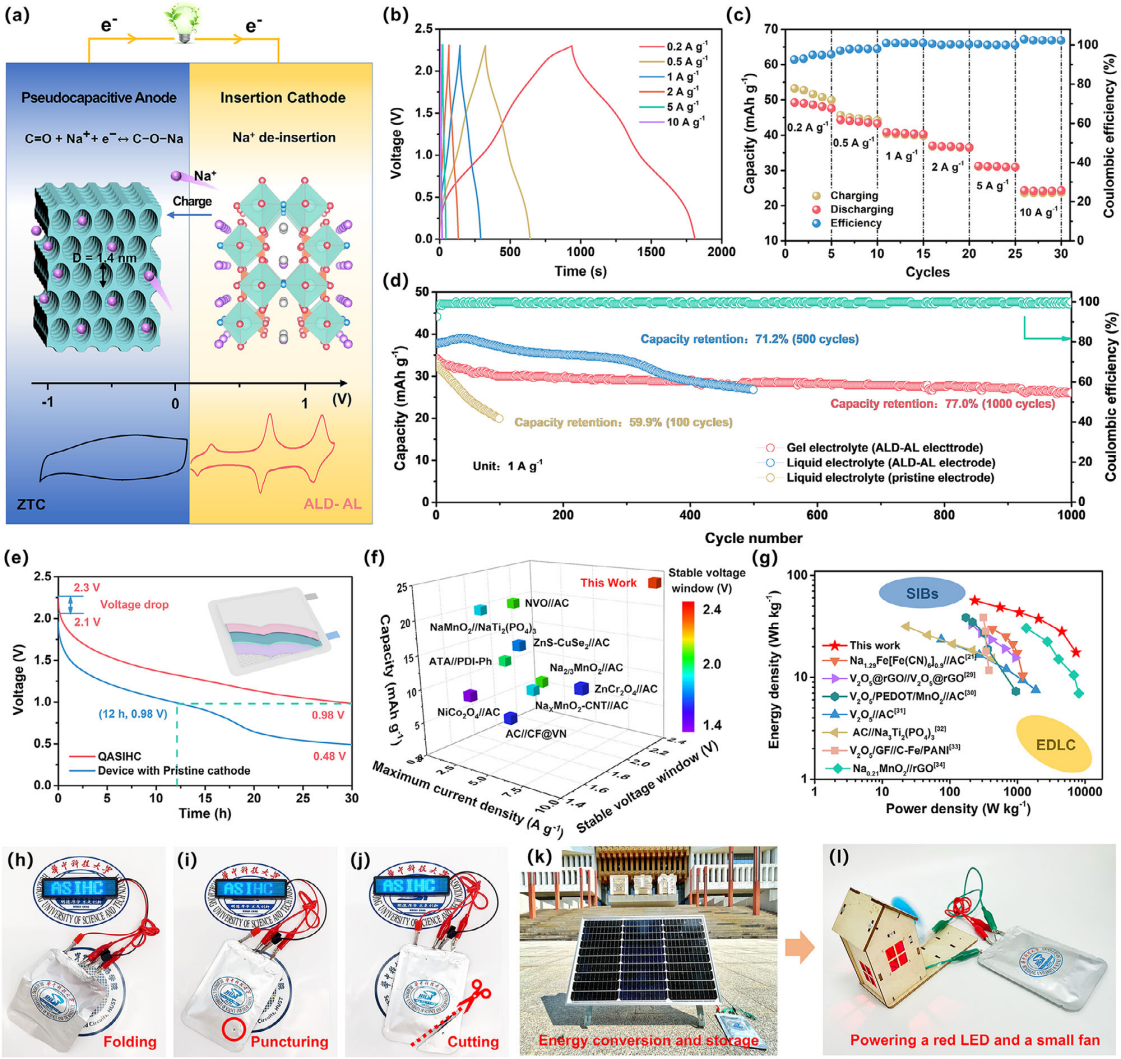
a) Schematic illustration of our device configuration and energy storage mechanism. b) GCD curves at various current densities. c) Rate performance. d) Cycle stability and coulombic efficiency comparison using different cathodes and electrolytes. e) Comparison of self‐discharge curves. f) Comparison of rate capability and stable voltage window of QASIHC with recent literature; more details are provided in Table S5 (Supporting Information). g) Ragone plot. Optical pictures of the pouch‐type QASIHC device powering a digital screen after h) folding, i) puncturing, j) cutting. Demonstration of QASIHC k) being charged from solar panels in the daytime, and l) then powering a red LED and a small fan at night. The logos owned by the relevant university were used with permission. The electrochemical data of devices were all estimated based on the total active mass of the cathode and anode.

Our ALD‐AL cathode and ZTC anode can be coupled to construct a unique high‐voltage ASIHC (**Figure** **5**a). Based on the half‐cell tests, the mass ratio of the cathode to anode is set as 1.1:1 to realize the charge balance. Figure S32 (Supporting Information) depicts the CV profiles of our ALD‐AL//ZTC ASIHC at various scan rates, showing a large voltage window of ≈2.3 V that is superior to most of the reported ASIHCs.^[^ ^27^ ^]^ The ASIHC can be effectively discharged at current densities from 0.2 to 10 A g^−1^; all the GCD curves display a quasi‐triangular shape with very sloping plateaus, characteristic of a typical hybrid energy storage mechanism (Figure 5b). Our device delivers a reversible capacity of ≈50 mAh g^−1^ at 0.2 A g^−1^ (based on the total active mass of the cathode and anode); even with increasing the current density 50 times to 10 A g^−1^, ≈50% of the capacity at 0.2 A g^−1^ can still be retained (Figure 5c), indicating outstanding rate capability.

However, using liquid electrolytes inevitably results in safety hazards, such as electrolyte leakage and solvent evaporation. For more sustainable application purposes, we further designed a polyacrylamide (PAM)‐based hydrogel polymer electrolyte to develop a quasi‐solid‐state ASIHC device (denoted as QASIHC; Figure S33, Supporting Information). PAM was chosen because it generally possesses superior compatibility and mechanical robustness in comparison to other polymers when designing hydrogel electrolytes. The QASIHC demonstrates very similar GCD profiles to ALD‐AL//ZTC ASIHC with only slightly smaller capacities (Figure S34, Supporting Information), which is probably due to the fact that the solid/quasi‐solid interfacial contact is not as perfect as the general solid/liquid contact.^[^ ^28^ ^]^ Despite this, our QASIHC still exhibits an excellent rate capability (Figure S35, Supporting Information), with a capacity retention rate comparable to that of the ALD‐AL//ZTC ASIHC. The cycling performance of three purpose‐designed devices is further shown in Figure 5d for comparison. Compared with the device using pristine cathode and liquid electrolyte, the device using ALD‐AL cathode and liquid electrolyte shows superior cycling stability (100 cycles, 59.9% vs 500 cycles, 71.2%); this once again highlights the importance of ALD precoating on the operation stability of NVOPF. Notably, the QASIHC possesses a 77.0% capacity retention after even much longer cycles of 1000 times; the further improved cycling performance implies that the hydrogel electrolyte also stabilizes the cathode materials by inhibiting dissolution and diffusion.^[^ ^29^ ^]^ The cycling stability is expected to be further improved in the future via electrolyte regulation to suppress the water activity and form a more robust interphase. The rate of self‐discharge (SDC) is an important indicator of energy storage devices including hybrid capacitors. Thus, the effect of in situ reconstructed AlF_3_‐rich interphase on the SDC property was investigated (Figure 5e). Notably, the SDC process of our QASIHC is much slower than that of the device using pristine cathode (without ALD precoating); after only 12 h the voltage of the device using pristine cathode already decreases to 0.98 V, whereas it needs to take 30 h for QASIHC.

Figure 5f and Table S5 (Supporting Information) further compare the stable voltage window and rate capability of our device with those reported in the literature. As can be seen, conventional aqueous hybrid capacitors typically show a narrower voltage window (<2.0 V) and inferior capacities at high current densities; by contrast, our device far surpasses most of the reported ASIHCs and even some aqueous sodium‐ion batteries such as Na_3_V_2_(PO_4_)_3_//NaTi_2_(PO_4_)_3_^[^ ^30^ ^]^ in terms of these attributes due to elaborate configuration design. Figure 5g illustrates the Ragone plot of gravimetric energy density versus the power density of our device, including data from previous sodium‐ion energy storage systems for comparison. In general, the gravimetric energy density of our ASIHC (56.6 Wh kg^−1^ at 233.4 W kg^−1^; 17.6 Wh kg^−1^ at 7351.2 W kg^−1^) are much larger than many reported ASIHCs such as Na_1.29_Fe[Fe(CN)_6_]_0.9_//AC (23.5 Wh kg^−1^ at 4424.7 W kg^−1^),^[^ ^23^ ^]^ V_2_O_5_@rGO//V_2_O_5_@rGO (27.2 Wh kg^−1^ at 216.1 W kg^−1^),^[^ ^31^ ^]^ V_2_O_5_/PEDOT/MnO_2_//AC (38.5 Wh kg^−1^ at 172.2 W kg^−1^),^[^ ^32^ ^]^ V_2_O_5_//AC (16.6 Wh kg^−1^ at 272.1 W kg^−1^),^[^ ^33^ ^]^ AC//Na_3_Ti_2_(PO_4_)_3_ (18.4 Wh kg^−1^ at 215.3 W kg^−1^),^[^ ^34^ ^]^ V_2_O_5_/GF//C‐Fe/PANI (38.5 Wh kg^−1^ at 312.6 W kg^−1^)^[^ ^35^ ^]^ and Na_0.21_MnO_2_//rGO (17.3 Wh kg^−1^ at 6808.5 W kg^−1^).^[^ ^36^ ^]^ Especially, the high energy density even exceeds that of some typical aqueous sodium‐ion batteries such as Na_3_MnTi(PO_4_)_3_//Na_3_MnTi(PO_4_)_3_ (22.06 Wh kg^−1^),^[^ ^37^ ^]^ Na_X_MnO_2_//MoO_3_@PPy (20.67 Wh kg^−1^)^[^ ^38^ ^]^ and NaMnO_2_//NaTi_2_(PO_4_)_3_ (21.02 Wh kg^−1^).^[^ ^39^ ^]^

Considering that our hydrogel polymer electrolyte can not only serve as a separator to isolate the cathode and anode but also provide strong adhesion to electrodes,^[^ ^40^ ^]^ it is extremely convenient to assemble integrated pouch‐cell QASIHC. The QASIHC cells are also readily connected in parallel or in series to change the output current or voltage to meet the energy and power supply for practical applications (Figure S36, Supporting Information). Additionally, the pouch‐type QASIHC works well under extreme conditions, such as folding (Figure 5h), puncturing (Figure 5i), and cutting (Figure 5j), signifying its great safety in practical applications. The outstanding performance of our QASIHC motivated us to explore its application potential in residential decentralized photovoltaic systems. As displayed in Figure 5k, the QASIHC pouch cell assembled in a fashion of two in series is integrated with solar photovoltaic panels (size: 55 × 30 cm, maximum power: 30 W) for charging at daytime. When the energy conversion process of converting solar energy into electrochemical energy is finished, our QASIHC device can power a red LED and a small fan used in the house model (Figure 5l), which represent the lighting system and power equipment, respectively. The successful integration of our QASIHCs with photovoltaic panels module demonstrates their feasibility for application as a large‐scale energy storage technique in future smart/microgrids.

## 3 Conclusion

In summary, a novel approach for in situ constructing robust CEI on an F‐containing cathode in aqueous electrolyte is proposed. As a proof of concept, the conformal Al_2_O_3_ sacrificial layer is pre‐deposited on a fluorinated polyanionic cathode of NVOPF, which can progressively react with trace HF derivate to form dense interphase of AlF_3_ and NaAlO_2_ species during cycling. The unusual AlF_3_‐rich interphase contributes to mitigating interfacial side reactions, inhibiting vanadium dissolution, and promoting Na^+^ transport kinetics, leading to significantly enhanced electrochemical performance of NVOPF electrodes. By pairing with pseudocapacitive ZTC anode and PAM‐based hydrogel electrolyte, the resulting QASIHC also exhibits remarkable cycling stability, high energy/power densities, and exceptional safety, which further demonstrate its application potential in future smart/microgrids. This work provides a new perspective for interface engineering toward unstable cathodes in aqueous electrolytes and paves the way for designing low‐cost, high‐energy aqueous energy storage systems.

## 4 Experimental Section

### Synthesis of NVOPF@rGO Composite

NVOPF@rGO composite was synthesized using a facile redox hydrothermal method developed by the group.^[^ ^15^ ^]^ Specifically, 359.0 mg Vanadium (III) acetylacetonate, 104 µL H_3_PO_4_, and 71.5 mg NaF were first dissolved in 3 mL ethanol and 1 mL acetone. Subsequently, 4.5 g 1 wt.% GO aqueous suspension was added to the above solution and stirred for 10 min. The mixture was then transferred into 25 mL Teflon‐line stainless steel autoclave and heated at 120 °C for 10 h to allow the interfacial redox reaction between V^3+^ precursor and GO. Finally, the obtained grayish‐black powder was washed repeatedly with deionized water and ethanol and dried in a hot‐air oven at 60 °C for 12 h.

### Preparation of ALD‐AL Cathode

Typically, a mixture of NVOPF@rGO (60 wt%), super P carbon black (20 wt.%), and polytetrafluoroethylene (PTFE, 20 wt.%) was pressed onto Ti mesh under the pressure of 15 MPa. The active material loading was controlled at ≈4 mg cm^−2^. Furthermore, the as‐prepared NVOPF@rGO electrode was deposited with Al_2_O_3_ by ALD instrument (Picosun SUNALE R‐200). In detail, Trimethyl aluminum (TMA) and H_2_O were used as precursors. During the deposition process, the temperature of the reaction chamber was maintained at 150 °C. Each ALD cycle consisted of a 0.2 s precursor pulse and 10 s purging time with Ar gas. The thickness of the deposited Al_2_O_3_ layer was precisely controlled via cycle number (≈1.4 Å per cycle).

### Synthesis of ZTC Anode

ZTC was synthesized by a well‐established technique, using zeolite as the template.^[^ ^41^ ^]^ First, furfuryl alcohol was infused into the pores of the zeolite under reduced pressure. Then, the resulting composite was heated at 5 °C min^−1^ under N_2_ flow. When the temperature reached 900 °C, chemical‐vapor deposition (CVD) of propylene was carried out at 700 °C for 2 h. The obtained materials were subsequently heated under N_2_ flow at 900 °C for 3 h. Finally, the zeolite template was dissolved by HF treatment (47% aqueous solution), and the obtained sediment was washed with distilled water and dried overnight at 120 °C to get the ZTC powder. For the preparation of the ZTC anode, a mixture of ZTC (80 wt.%), super P carbon black (10 wt.%), and polytetrafluoroethylene (PTFE, 10 wt.%) was pressed onto Ti mesh under the pressure of 15 MPa.

### Assembly of ALD‐AL//ZTC Hybrid Capacitor

The PAM‐17 m NaClO_4_ hydrogel electrolyte was prepared as follows: 1 g acrylamide (AM) was first mixed with 10 mL 17 m NaClO_4_ aqueous solution, then 5 mg N, N’‐methylene‐bis(acrylamide) (MBA) and 10 mg K_2_S_2_O_8_ were added into the above solution and heated at 60 °C for 4 h. To fabricate the quasi‐solid‐state hybrid device, ALD‐AL cathode, and ZTC anode were coated with PAM‐17 m NaClO_4_ sol and then assembled face to face for gelation.

### Characterizations

The morphology, composition, and crystalline structure of as‐prepared samples were characterized by using SEM (Gemini SEM 300) with EDS (X‐Max 50), AFM (Bruker Dimension Icon), TEM (JEM‐F200; 200 kV), XRD with Cu Kα radiation (Bruker D‐8 Avance), ATR‐FTIR (Thermo scientific iS50), and Raman spectroscopy (LabRAM HR Evolution (532)). The surface chemical composition and valence states were determined by XPS (ESCALAB 250Xi, USA). The vanadium content was analyzed by AAS (contrAA700, Germany). The interphase components of the cycled ALD‐AL electrode were analyzed by a TOF.SIMS 5 spectrometer (ION‐TOF Gmhb 5). The pH value of electrolytes was measured by a pH meter (PHS‐3C), and the F^−^ content was determined by ion‐selective electrode analysis.

### Electrochemical Measurements

All the electrochemical measurements were carried out using a CS2350H electrochemical workstation. For individual working electrodes, the electrochemical tests were conducted in a three‐electrode mode using SCE as reference electrode and Pt foil as counter electrode in 17 m NaClO_4_ electrolyte. The specific capacities were calculated from GCD curves by using the equation: *Q*_s_ =  *It*/*m*, where *I* is the discharging current (A), *t* is the total discharging time (s), *m* is the mass of active materials (g). The gravimetric energy and power densities (*E* and *P*) were estimated based on E=∫IV(t)dt and *P* = *E*/Δ*t*, respectively; where *I* is the discharging current density (A g^−1^), *V*(*t*) is discharging voltage at *t* (V), d*t* is the time differential, and Δ*t* is the total discharging time (s).

### Computational Methods

The spin‐polarized DFT calculations were carried out in the Vienna ab initio simulation package (VASP) based on the projector augmented‐wave (PAW) method.^[^ ^42^ ^]^ The exchange‐correlation potential was treated by using a generalized gradient approximation (GGA) with the Perdew‐Burke‐Ernzerhof (PBE) parametrization.^[^ ^43^ ^]^ The cutoff energy of atomic wave functions was set to be 400 eV, and the Brillouin‐zone integration was sampled with a Γ‐centered Monkhorst–Pack mesh of 2 × 2 × 1 by VASPKIT. In the calculation process, the structures were fully relaxed until the maximum force on each atom was less than 0.05 eV Å^−1^, and the energy convergent standard was 10^−4^ eV. The van der Waals correction of Grimme's DFT‐D3 model was also adopted.^[^ ^44^ ^]^ To avoid the periodic interactions for interface structures, a vacuum layer as large as 15 Å was used along the *c* direction normal to the interface. The amorphous models were built by ab initio molecular dynamics with the canonical NVT ensemble for 10 ps by a timestep of 1 fs at T = 4000 K and 3000 K for Al_2_O_3_ and AlF_3_, respectively. The corresponding migration energy barriers of Na^+^ were calculated through the nudged elastic band (CINEB) method.^[^ ^45^ ^]^

## Conflict of Interest

The authors declare no conflict of interest.

## Supporting information



Supporting Information

## Data Availability

The data that support the findings of this study are available from the corresponding author upon reasonable request.

## References

[1] a) S. S. Cheema , N. Shanker , S. L. Hsu , J. Schaadt , N. M. Ellis , M. Cook , R. Rastogi , R. N. Podgurski , J. Ciston , M. Mohamed , S. Salahuddin , Nature 2024, 629, 803; 38593860 10.1038/s41586-024-07365-5

[2] a) H. Wu , J. Hao , Y. Jiang , Y. Jiao , J. Liu , X. Xu , K. Davey , C. Wang , S. Qiao , Nat. Commun. 2024, 15, 575; 38233408 10.1038/s41467-024-44855-6 PMC10794691

[3] a) H. Zhang , X. Tan , H. Li , S. Passerini , W. Huang , Energy Environ. Sci. 2021, 14, 5788;

[4] a) S. Xu , Y. Yang , F. Tang , Y. Yao , X. Lu , L. Liu , C. Xu , Y. Feng , X. Rui , Y. Yu , Mater. Horiz. 2023, 10, 1901; 36942608 10.1039/d3mh00003f

[5] Y. Dai , C. Zhang , J. Li , X. Gao , P. Hu , C. Ye , H. He , J. Zhu , W. Zhang , R. Chen , W. Zong , F. Guo , I. P. Parkin , D. L. Brett , P. R. Shearing , L. Mai , G. He , Adv. Mater. 2024, 36, 2310645. 38226766 10.1002/adma.202310645 PMC11475447

[6] a) K. Wu , Z. Li , X. Chen , Adv. Funct. Mater. 2024, 34, 2315327;

[7] S. Liu , L. Wang , J. Liu , M. Zhou , Q. Nian , Y. Feng , Z. Tao , L. Shao , J. Mater. Chem. A 2019, 7, 248.

[8] P. R. Kumar , Y. H. Jung , D. K. Kim , J. Solid State Electr. 2017, 21, 223.

[9] G. Su , Y. Wang , J. Mu , Y. Ren , P. Yue , W. Ji , L. Liang , L. Hou , M. Chen , C. Yuan , Adv. Energy Mater. 2024, 15, 2403282.

[10] P. Guan , L. Zhou , Z. Yu , Y. Sun , Y. Liu , F. Wu , Y. Jiang , D. Chu , J. Energy Chem. 2020, 43, 220.

[11] a) N. Zhang , B. Wang , F. Jin , Y. Chen , Y. Jiang , C. Bao , J. Tian , J. Wang , R. Xu , Y. Li , Q. Lv , H. Ren , D. Wang , H. Liu , S. Dou , X. Hong , Cell Rep. Phys. Sci. 2022, 3, 101197;

[12] J. C. Hestenes , L. E. Marbella , ACS Energy Lett. 2023, 8, 4572.

[13] a) F. Wang , Y. Lin , L. Suo , X. Fan , T. Gao , C. Yang , F. Han , Y. Qi , K. Xu , C. Wang , Energy Environ. Sci. 2016, 9, 3666;

[14] a) Y. Li , Y. Yang , P. Zhou , T. Gao , Z. Xu , S. Lin , H. Chen , J. Zhou , S. Guo , Matter 2019, 1, 893;

[15] D. Ba , Q. Gui , W. Liu , Z. Wang , Y. Li , J. Liu , Nano Energy 2022, 94, 106918.

[16] Y. Wang , J. Liu , T. Chen , W. Lin , J. Zheng , Phys. Chem. Chem. Phys. 2022, 24, 2167. 35005758 10.1039/d1cp04049a

[17] a) X. Lu , S. Li , Y. Li , F. Wu , C. Wu , Y. Bai , Adv. Mater. 2024, 36, 2407359; 10.1002/adma.202407359 38936413

[18] P. Gong , J. Xia , C. Chen , Z. Zhao , D. Liu , Y. Li , J. Liu , Chem. Eng. J. 2024, 495, 153445.

[19] N. R. Lashari , M. Zhao , J. Wang , X. He , I. Ahmed , M. Liang , S. Tangsee , X. Song , Energy Fuel 2021, 35, 20394.

[20] A. A. Nechikott , H. D. Yoo , P. K. Nayak , J. Power Sources 2024, 591, 233825.

[21] J. Han , M. Zarrabeitia , A. Mariani , Z. Jusys , M. Hekmatfar , H. Zhang , D. Geiger , U. Kaiser , R. J. Behm , A. Varzi , S. Passerini , Nano Energy 2020, 77, 105176.

[22] W. Song , X. Ji , Y. Zhu , H. Zhu , F. Li , J. Chen , F. Lu , Y. Yao , C. E. Banks , ChemElectroChem 2014, 1, 871.

[23] L. Zhou , Z. Yang , C. Li , B. Chen , Y. Wang , L. Fu , Y. Zhu , X. Liu , Y. Wu , RSC Adv. 2016, 6, 109340.

[24] J. Zhang , D. Zhang , F. Niu , X. Li , C. Wang , J. Yang , ChemPlusChem 2017, 82, 1170. 31957293 10.1002/cplu.201700258

[25] A. Tron , Y. D. Park , J. Mun , J. Power Sources 2016, 325, 360.

[26] W. Y. Liu , W. J. Cui , C. J. Yi , J. L. Xia , J. B. Shang , W. F. Hu , Z. Wang , X. H. Sang , Y. Y. Li , J. P. Liu , Nat. Commun. 2024, 15, 9889. 39543206 10.1038/s41467-024-54317-8 PMC11564968

[27] a) Q. Chen , J. Jin , M. Song , X. Zhang , H. Li , J. Zhang , G. Hou , Y. Tang , L. Mai , L. Zhou , Adv. Mater. 2022, 34, 2107992; 10.1002/adma.202107992 34882849

[28] J. Liu , C. Guan , C. Zhou , Z. Fan , Q. Ke , G. Zhang , C. Liu , J. Wang , Adv. Mater. 2016, 28, 8732. 27562134 10.1002/adma.201603038

[29] Y. Jiang , D. Ba , Y. Li , J. Liu , Adv. Sci. 2020, 7, 1902795. 10.1002/advs.201902795 PMC7080538 32195094

[30] Q. Zhang , C. Liao , T. Zhai , H. Li , Electrochim. Acta 2016, 196, 470.

[31] H. Liu , W. Zhu , D. Long , J. Zhu , G. Pezzotti , Appl. Surf. Sci. 2019, 478, 383.

[32] C. X. Guo , G. Yilmaz , S. Chen , S. Chen , X. Lu , Nano Energy 2015, 12, 76.

[33] Q. Qu , Y. Zhu , X. Gao , Y. Wu , Adv. Energy Mater. 2012, 2, 950.

[34] S. Zhang , Y. Liu , Q. Han , S. He , N. Zhang , J. Yang , J. Alloy. Compd. 2017, 729, 850.

[35] N. M. Ndiaye , B. D. Ngom , N. F. Sylla , T. M. Masikhwa , M. J. Madito , D. Momodu , T. Ntsoane , N. Manyala , J. Colloid. Interf. Sci. 2018, 532, 395. 10.1016/j.jcis.2018.08.010 30099303

[36] N. Karikalan , C. Karuppiah , S. Chen , M. Velmurugan , P. Gnanaprakasam , Chem.–Eur. J. 2017, 23, 2379. 27925320 10.1002/chem.201604878

[37] H. Gao , J. B. Goodenough , Angew. Chem., Int. Ed. 2016, 55, 12768. 10.1002/anie.201606508 27619012

[38] Y. Liu , B. H. Zhang , S. Y. Xiao , L. L. Liu , Z. B. Wen , Y. P. Wu , Electrochim. Acta 2014, 116, 512.

[39] Z. Hou , X. Li , J. Liang , Y. Zhu , Y. Qian , J. Mater. Chem. A 2015, 3, 1400.

[40] a) Q. Gui , W. Cui , D. Ba , X. Sang , Y. Li , J. Liu , Angew. Chem., Int. Ed. 2024, 63, 202409098; 10.1002/anie.202409098 39115086

[41] H. Lu , K. Kim , Y. Kwon , X. Sun , R. Ryoo , X. S. Zhao , J. Mater. Chem. A 2018, 6, 10388.

[42] a) P. Hohenberg , W. Kohn , Phys. Rev. 1964, 136, B864;

[43] J. P. Perdew , K. Burke , M. Ernzerhof , Phys. Rev. Lett. 1996, 77, 3865. 10062328 10.1103/PhysRevLett.77.3865

[44] S. Grimme , J. Antony , S. Ehrlich , H. Krieg , J. Chem. Phys. 2010, 132, 154104. 20423165 10.1063/1.3382344

[45] G. Henkelman , H. Jónsson , J. Chem. Phys. 2000, 113, 9978.

