# Supplementary material for: In Situ Converting Conformal Sacrificial Layer Into Robust Interphase Stabilizes Fluorinated Polyanionic Cathodes for Aqueous Sodium‐Ion Storage
Source: Adv Sci (Weinh). 2025 May 2;12(25):2501362. doi: 10.1002/advs.202501362 (PMC12224926; doi:10.1002/advs.202501362)
Supplement: Supplementary file 1 — Supporting Information [file ADVS-12-2501362-s001.pdf]

ADVANCED SCIENCE
Open Access

Supporting Information

for *Adv. Sci.*, DOI 10.1002/advs.202501362

In Situ Converting Conformal Sacrificial Layer Into Robust Interphase Stabilizes Fluorinated Polyanionic Cathodes for Aqueous Sodium-Ion Storage

Peng Gong, Shibo Chai, Xingjie Li, Yibo Dong, Shengjun Zhai, Xihao Chen, Ning Wang, Yuanyuan Li and Jinping Liu**

Supporting Information

In-Situ Converting Conformal Sacrificial Layer into Robust Interphase Stabilizes Fluorinated Polyanionic Cathodes for Aqueous Sodium-Ion Storage

Peng Gong, Shibo Chai, Xingjie Li, Yibo Dong, Shengjun Zhai, Xihao Chen, Ning Wang, Yuanyuan Li, and Jinping Liu**

P. Gong, Y. B. Dong, S. J. Zhai, Prof. Y. Y. Li
School of Integrated Circuits, Huazhong University of Science and Technology, Wuhan 430074, P. R. China
E-mail: liyynano@hust.edu.cn

P. Gong, S. B. Chai, X. J. Li, Prof. J. P. Liu
School of Chemistry, Chemical Engineering and Life Sciences and State Key Laboratory of Advanced Technology for Materials Synthesis and Processing, Wuhan University of Technology, Wuhan 430070, P. R. China
Email: liujp@whut.edu.cn

Dr. X. H. Chen
School of Materials Science and Engineering, Chongqing University of Arts and Sciences, Chongqing 402160, P. R. China

Dr. N. Wang
School of Science, Key Laboratory of High Performance Scientific Computation, Xihua University, Chengdu 610039, P. R. China

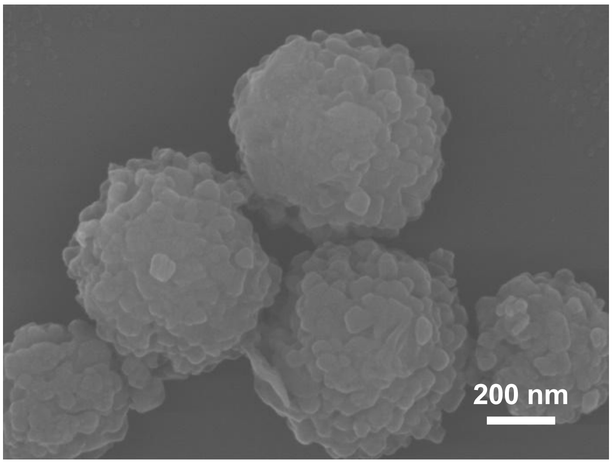

Figure S1. SEM image of NVOPF@rGO nanospheres.

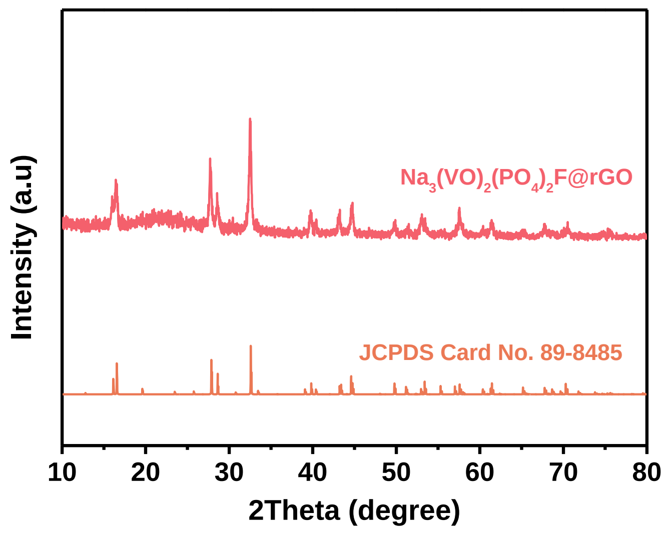

Figure S2. The XRD pattern of as-prepared NVOPF@rGO nanocomposites.

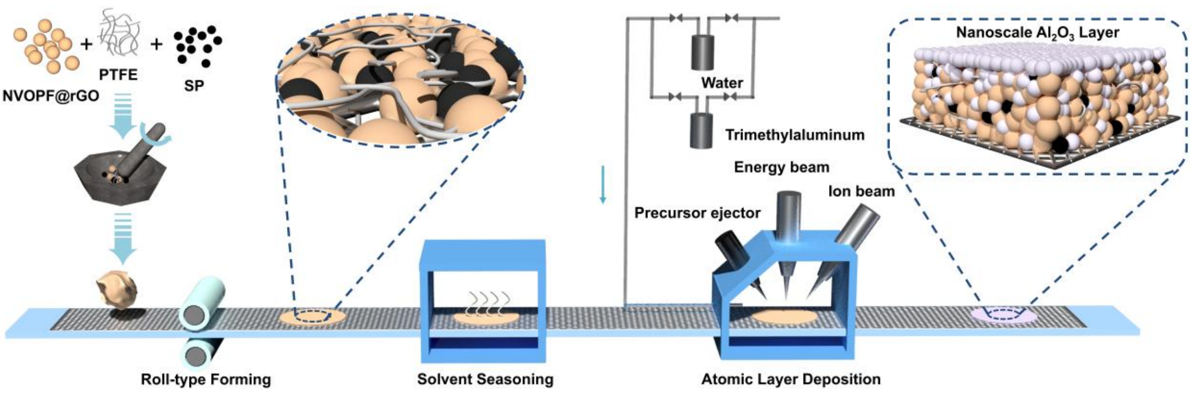

Figure S3. Schematic illustration of the preparation process of ALD-AL cathode.

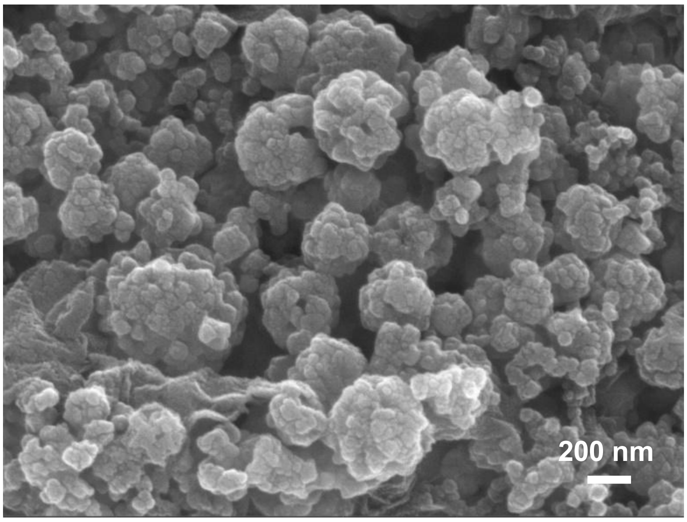

Figure S4. SEM image showing the surface of the pristine cathode.

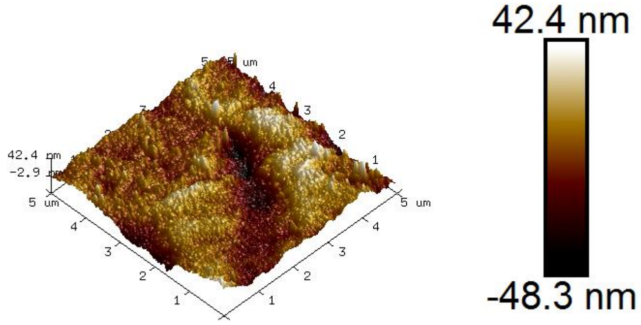

Figure S5. AFM image of the pristine cathode surface.

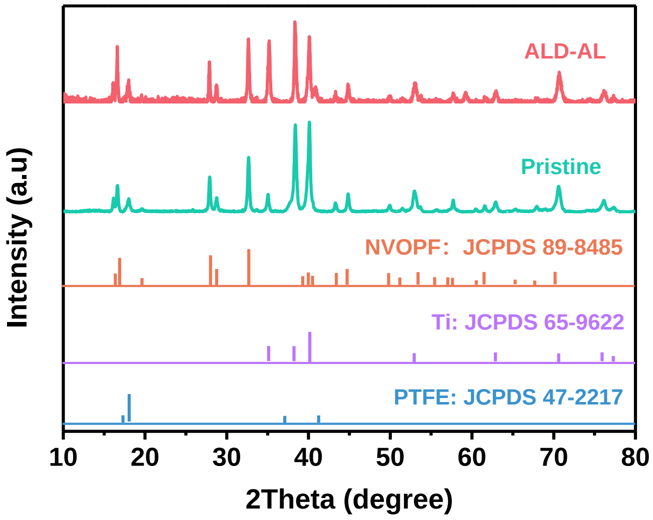

Figure S6. The XRD patterns of pristine cathode and ALD-AL cathode.

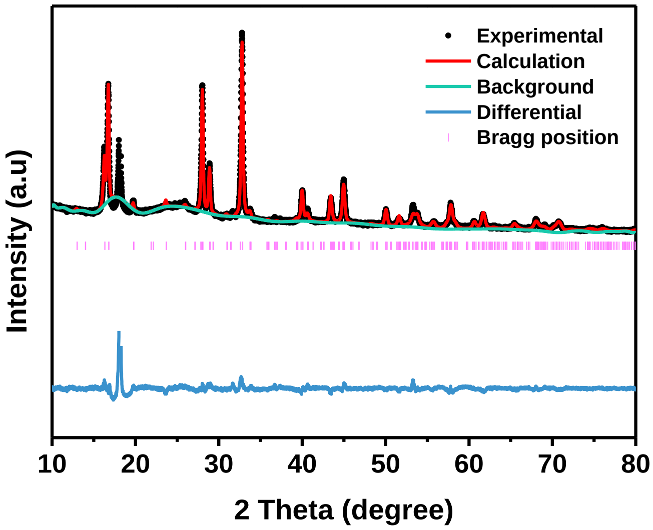

Figure S7. XRD Rietveld refinement of pristine cathode.

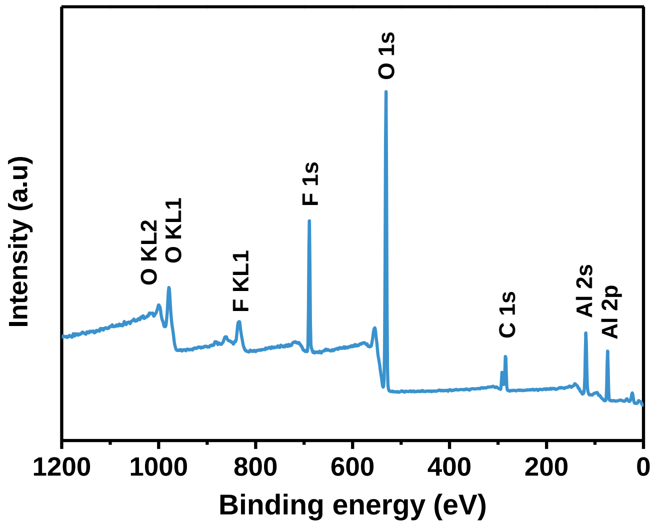

Figure S8. The XPS survey spectrum of the ALD-AL cathode.

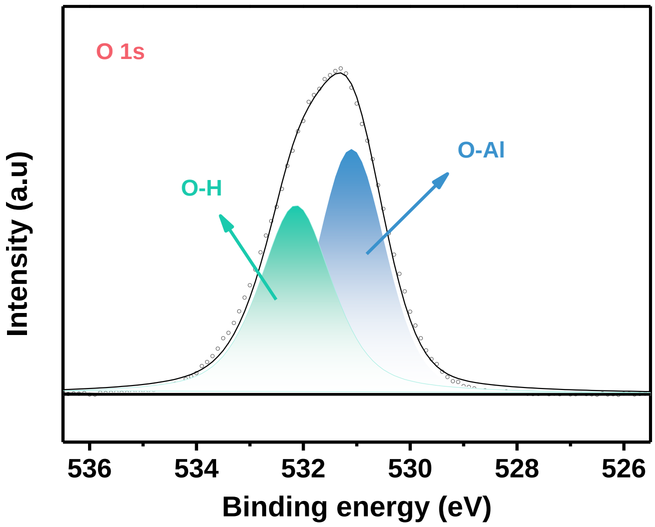

Figure S9. The high-resolution O 1s spectrum of ALD-AL cathode.

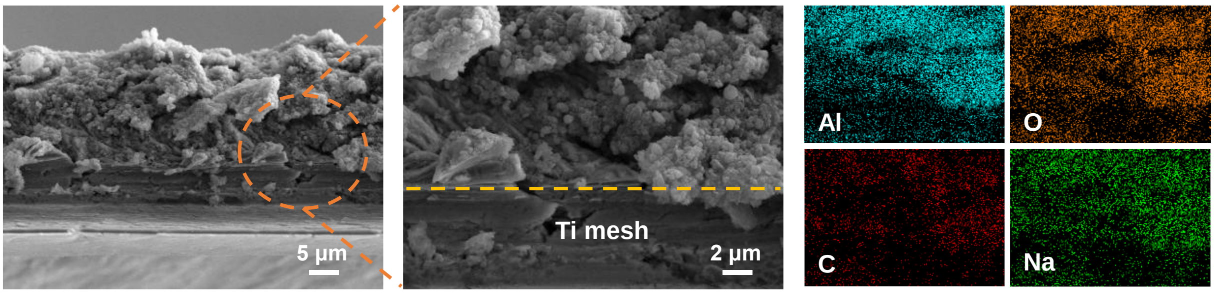

Figure S10. Cross-sectional SEM images of ALD-AL cathode and corresponding EDS mapping results.

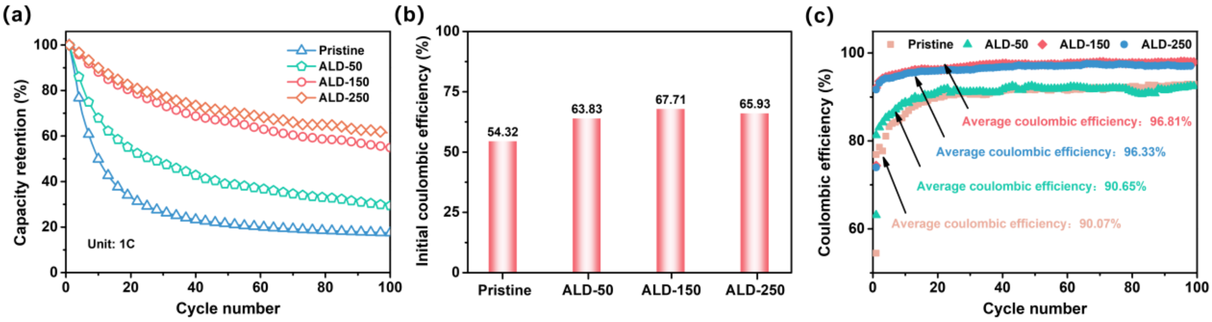

Figure S11. (a) Cycle stability comparison of pristine and ALD-*x* cathodes (*x* = 50, 150, and 250, representing ALD deposition cycles). (b) Initial coulombic efficiency comparison. (c) Average coulombic efficiency comparison.

Apparently, the thickness of Al_2O_3 layer (controlled by ALD deposition cycles) plays an important role in the overall performance of the cathode. With the optimal ALD deposition (150 cycles), the resulting ALD-AL cathode demonstrates superior comprehensive performance in terms of the cycling stability and coulombic efficiency; in this case, the in-situ generated interphase is believed to not only help stabilize the cathode, but also ensure relatively facile interfacial ion transport.

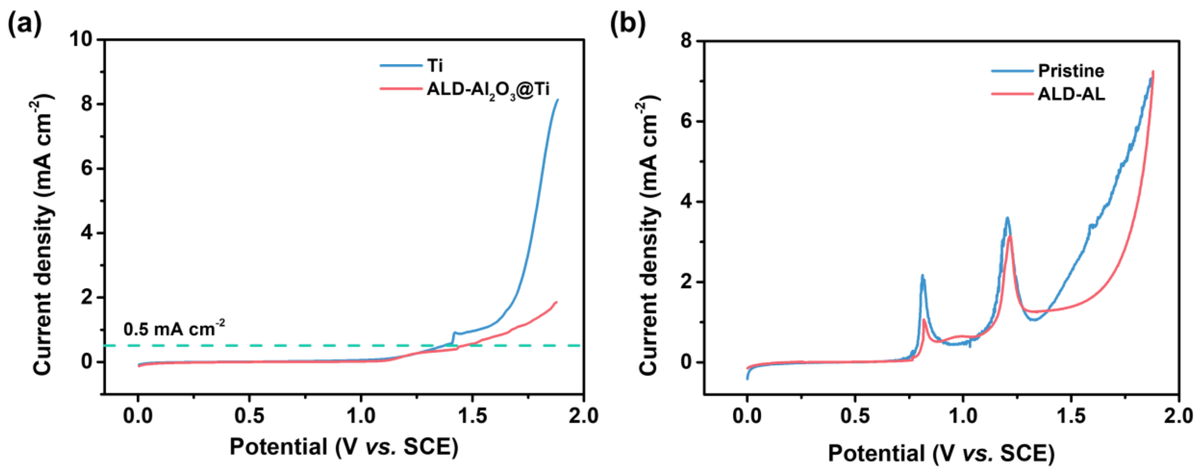

Figure S12. The linear sweep voltammetry (LSV) curves measured with (a) Ti-based electrodes and (b) practical electrodes.

Here, we used model electrodes to investigate the effect of Al_2O_3 layer on interfacial side reactions. Obviously, the measured electrochemical stability window on positive direction is extended, when the Ti electrode was coated by Al_2O_3, indicating that oxygen evolution reaction (OER) is indeed inhibited to some extent. This is because insulated Al_2O_3 layer separates electrode and electrolyte, preventing water molecules from obtaining electrons at the electrode surface. Similarly, the LSV profiles recorded using practical electrodes (i.e. the pristine and ALD-AL electrodes) also directly reveal the positive effect of Al_2O_3 on suppressing the interfacial side reactions. Note that the emerged two oxidation peaks are from the electrochemical Na^+ de-insertion reaction of NVOPF.

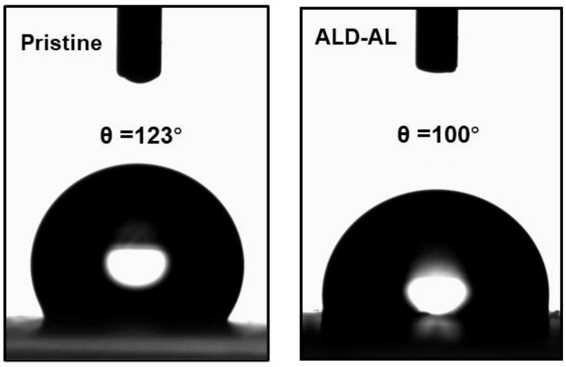

Figure S13. Contact angles of 17 m $NaClO_4$ electrolyte on pristine and ALD-AL cathodes.

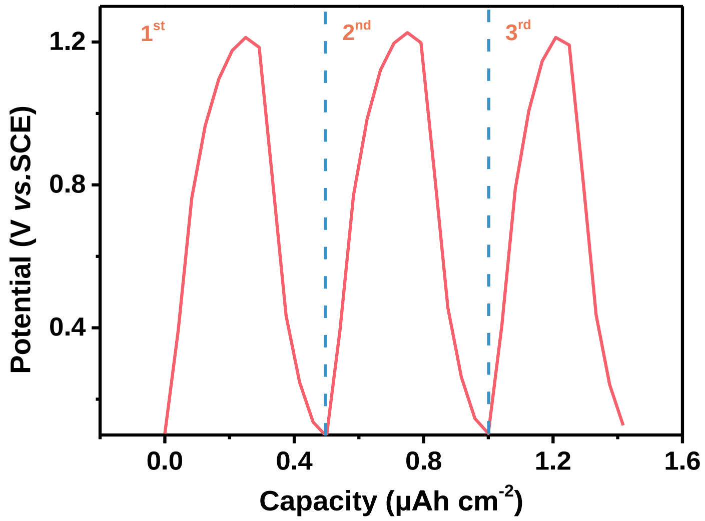

Figure S14. GCD curves of ALD-Al_2O_3@Ti model electrode at the initial three cycles.

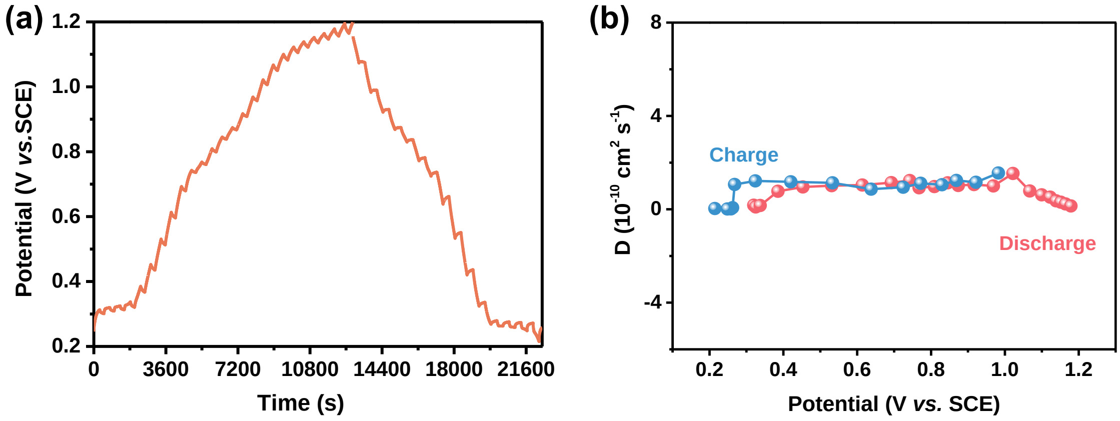

Figure S15. a) The galvanostatic intermittent titration technique (GITT) curves of ALD-AL cathode at the second cycle. b) Na^+ diffusion coefficients of ALD-AL cathode.

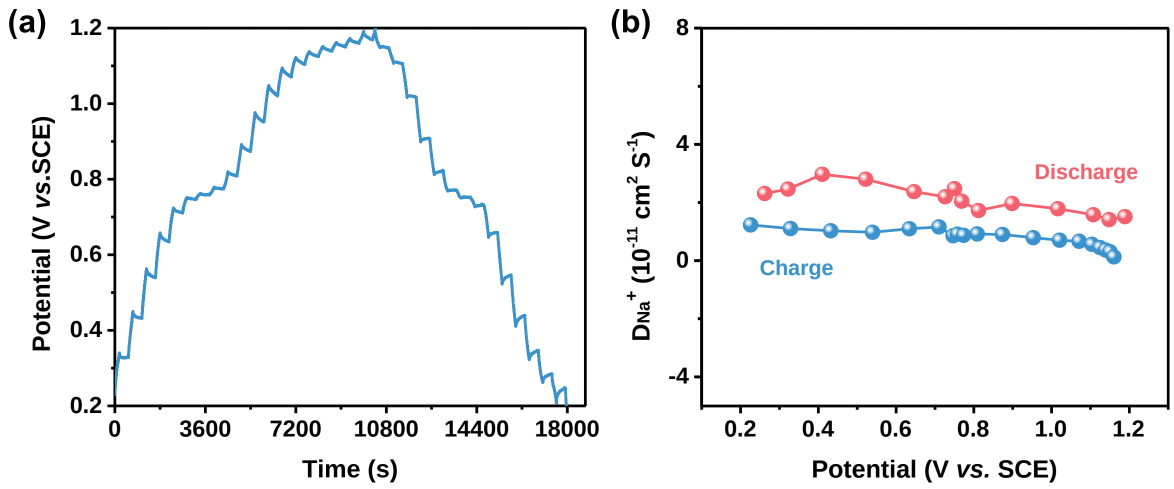

Figure S16. a) The galvanostatic intermittent titration technique (GITT) curves of pristine cathode at the second cycle. b) Na^+ diffusion coefficients of pristine cathode.

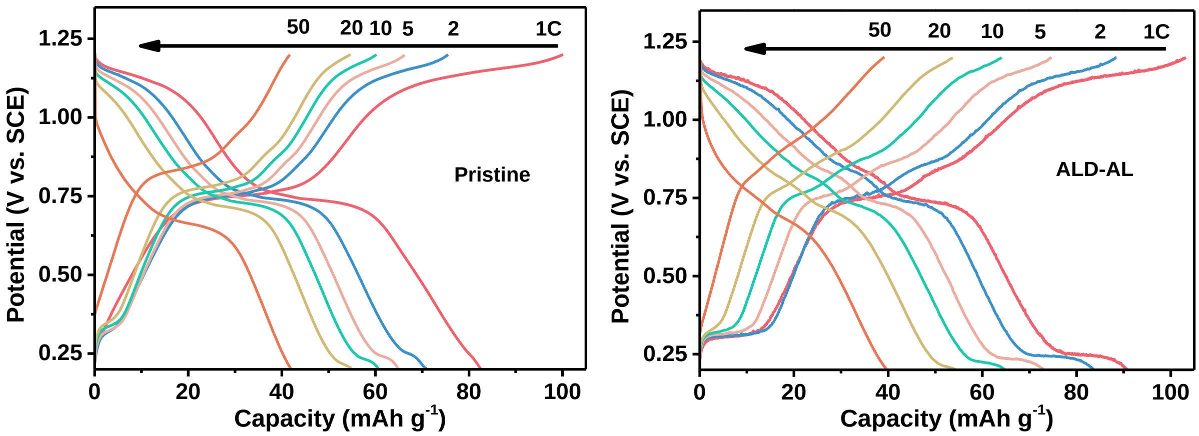

Figure S17. GCD curves of the pristine and ALD-AL cathodes at different C-rates.

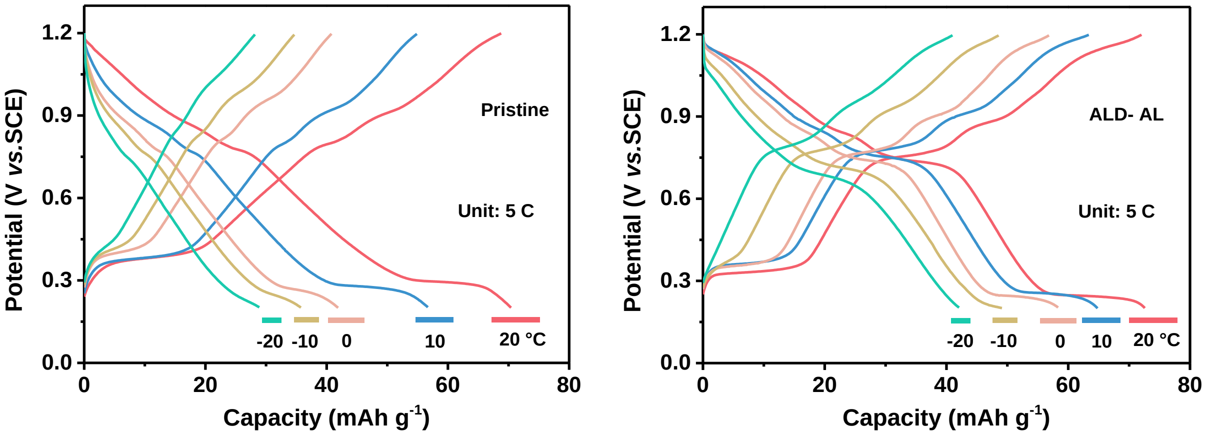

Figure S18. GCD curves of the pristine and ALD-AL cathodes at different temperatures.

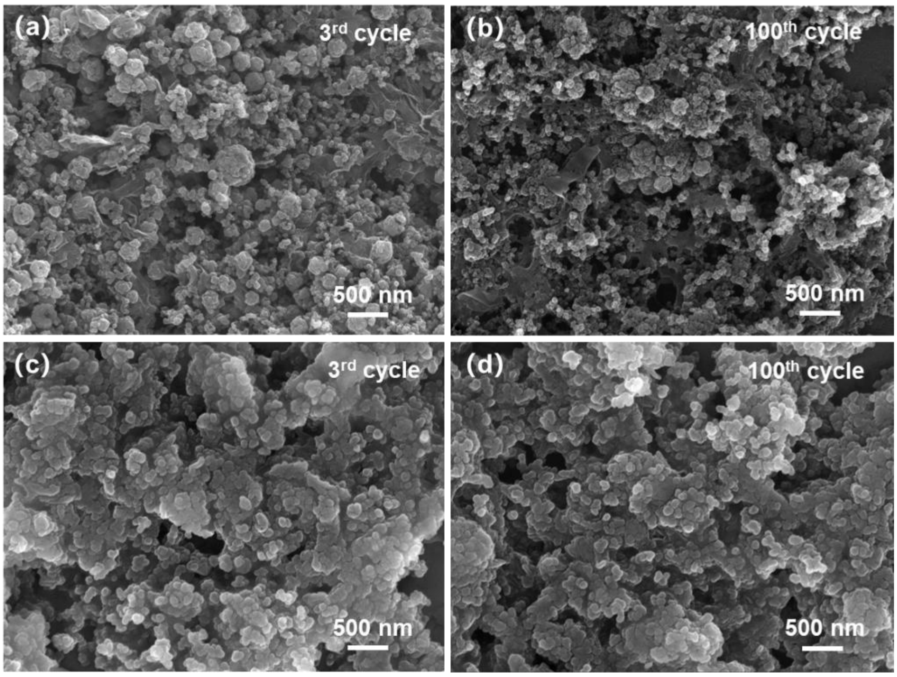

Figure S19. The top-view SEM images of (a, b) the pristine cathode and (c, d) ALD-AL cathode after 3 and 100 cycles.

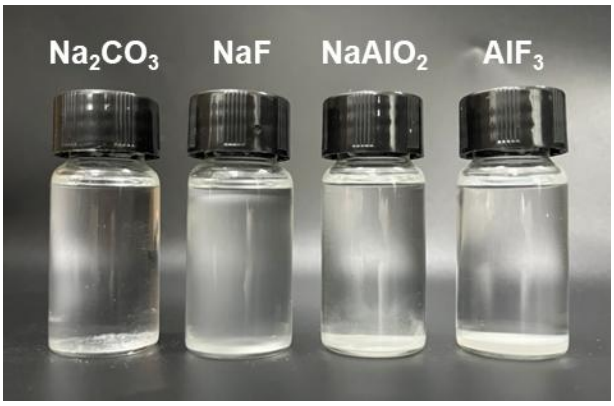

Figure S20. The optical photographs of 0.3 g Na_2CO_3, NaF, $NaAlO_2$, and AlF_3 in 10 mL 17 m $NaClO_4$ electrolyte, respectively.

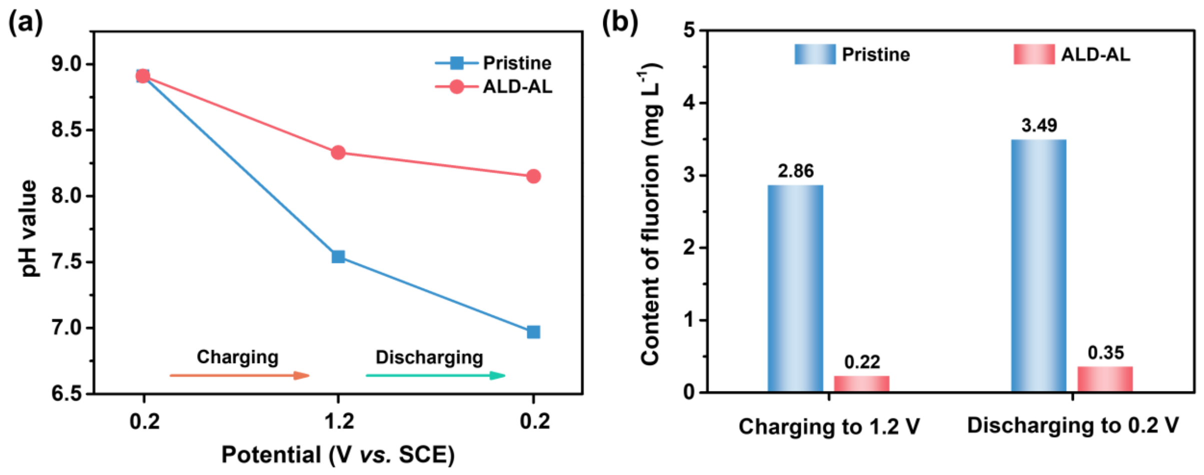

Figure S21. The variations of (a) pH value and (b) F^- content in the electrolyte during the first charge-discharge cycle.

For pristine cathode, the variations of pH and F^- content in electrolyte are obvious, directly indicating the generation of HF. For the ALD-AL cathode, the HF can still be detected, but the in-situ conversion of Al_2O_3 layer into AlF_3 interphase significantly consumes HF, leading to much smaller pH decrease and much lower F^- content in the electrolyte.

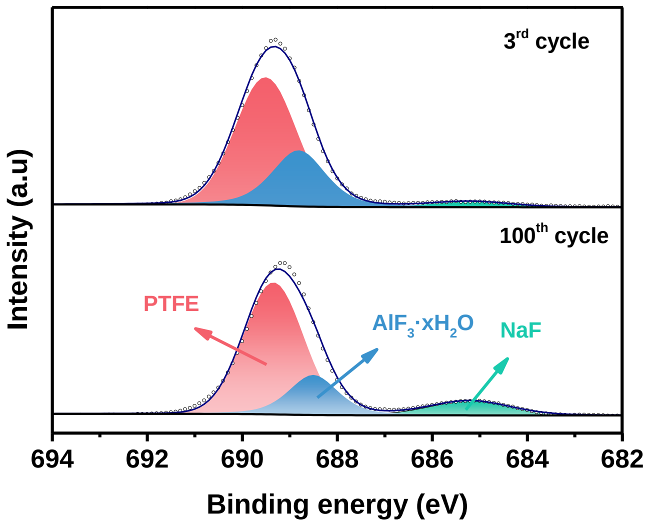

Figure S22. XPS high-resolution spectra of F 1s of the cycled ALD-AL cathode.

$$2H_2O \rightarrow O_2 + 2H^+ \quad \text{(electrolysis)}$$

$$Al_2O_3 + 6H^+ \rightarrow 2Al^{3+} + 3H_2O$$

$$Al^{3+} + 2H_2O \rightarrow AlO_2^- + 4H^+ \quad \text{(hydrolysis)}$$

Additionally, there is inevitably a small amount of NVOPF@rGO dissolving in the electrolyte during cycling, releasing a certain amount of Na^+ and F^-. Then, these free ions will combine with each other depositing on the electrode, including $NaAlO_2$, NaF, and AlF_3:

$$Na^+ + AlO_2^- \rightarrow NaAlO_2$$

$$Na^+ + F^- \rightarrow NaF$$

$$\mathbf{Al^{3+} + 3F^- \rightarrow AlF_3}$$

Meanwhile, trace HF is also generated. The pre-coating Al_2O_3 can react with harmful HF, converting to AlF_3:

$$H^+ + F^- \rightarrow HF$$

$$\mathbf{Al_2O_3 + 3HF \rightarrow AlF_3 \cdot xH_2O + (3-x)H_2O}$$

Figure S23. The possible interfacial reactions of Al_2O_3 interphase during cycling.

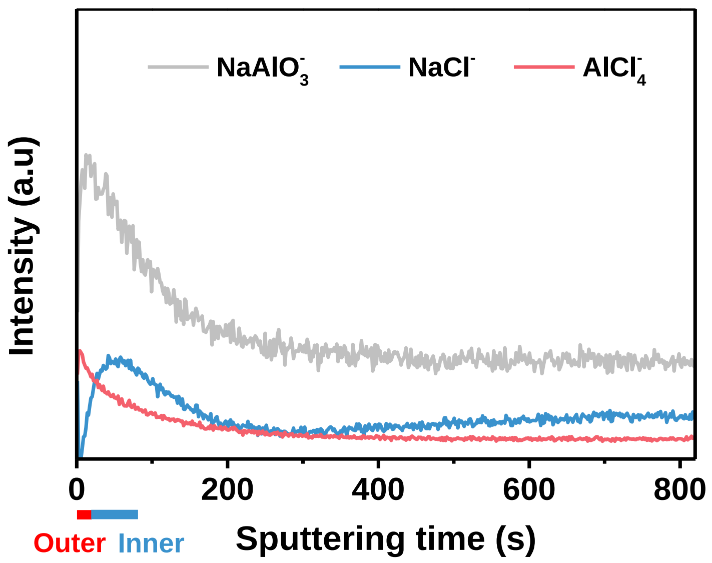

Figure S24. TOF-SIMS intensity depth profiles of chloride species and $NaAlO_3^-$.

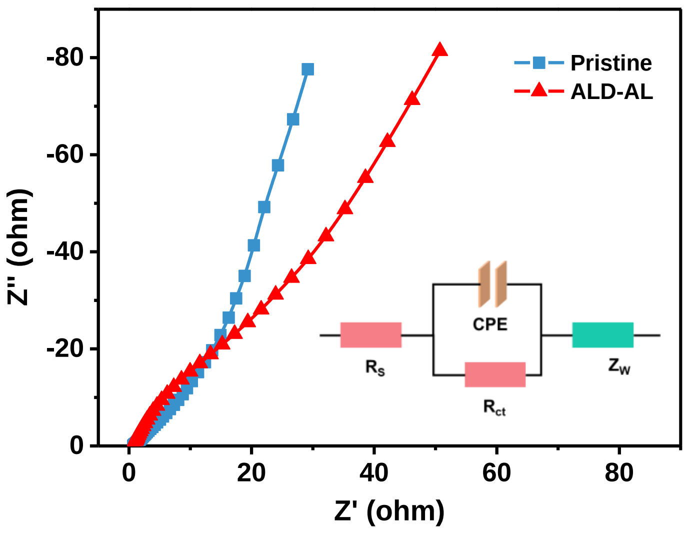

Figure S25. Nyquist plots of the pristine and ALD-AL cathodes before cycling.

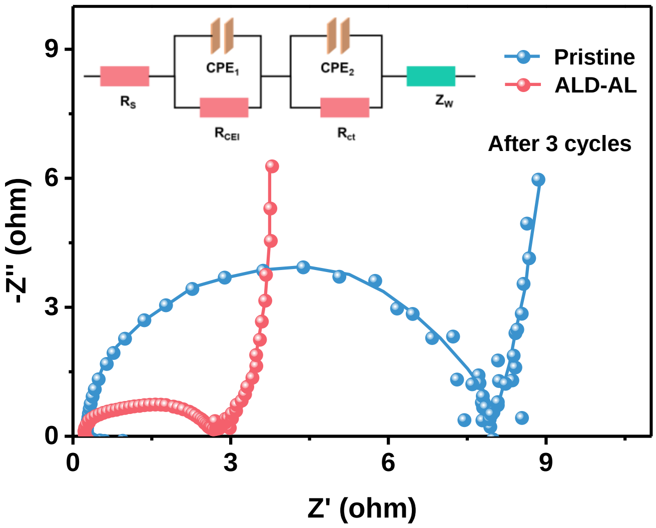

Figure S26. Nyquist plots of the pristine and ALD-AL cathodes after 3 cycles.

Sodium-ion diffusion coefficient (D_{Na+}) is a key parameter to evaluate the electrochemical reaction kinetics, which is calculated according to the following equations:

$$D_{Na^+}=R^2T^2/2A^2n^4F^4C^2\sigma_w^2 \qquad \text{equation (1)}$$

$$Z'=R_s+R_{ct}+\sigma_w\omega^{-1/2} \qquad \text{equation (2)}$$

where R is the gas constant; T is the absolute temperature; A is the electrode surface area; n is the number of electrons transfer per molecule, here n = 2; F is the Faraday constant; C is the concentration of Na^+; and σ_w is the Warburg impedance coefficient, which is evaluated according to equation (2). The specific values of σ_w are obtained by fitting linear relationship between real impedance (Z′) and the square root of angular frequency ($\omega^{-1/2}$), thus D_{Na+} can be calculated based on equation (1).

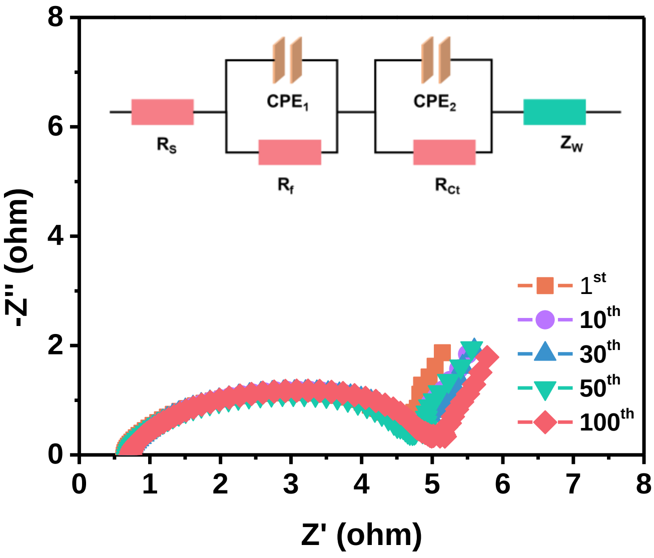

Figure S27. Nyquist plots of ALD-AL cathodes after different cycles.

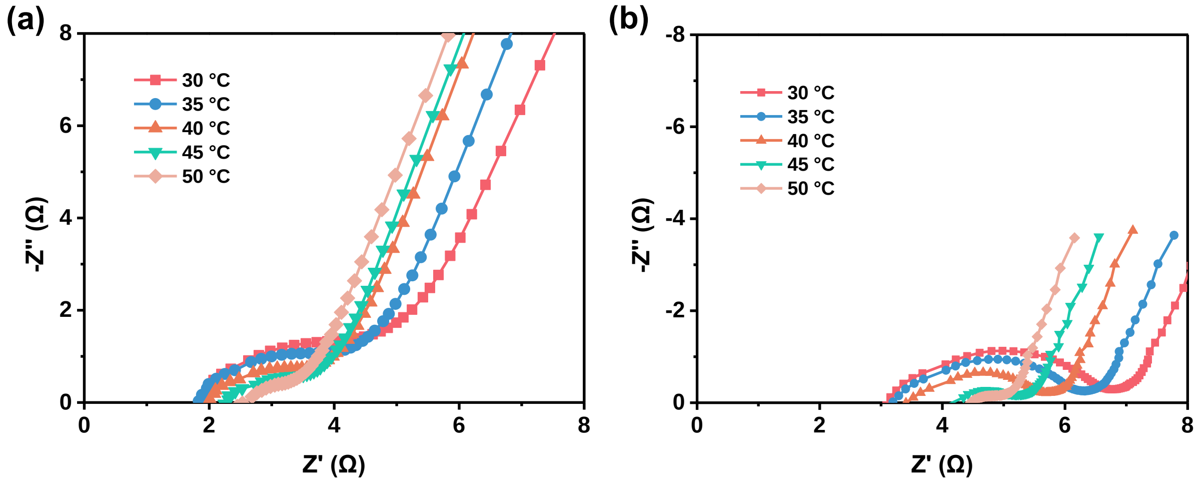

Figure S28. Nyquist plots of ALD-AL cathode (a) before and (b) after 3 cycles at different temperatures.

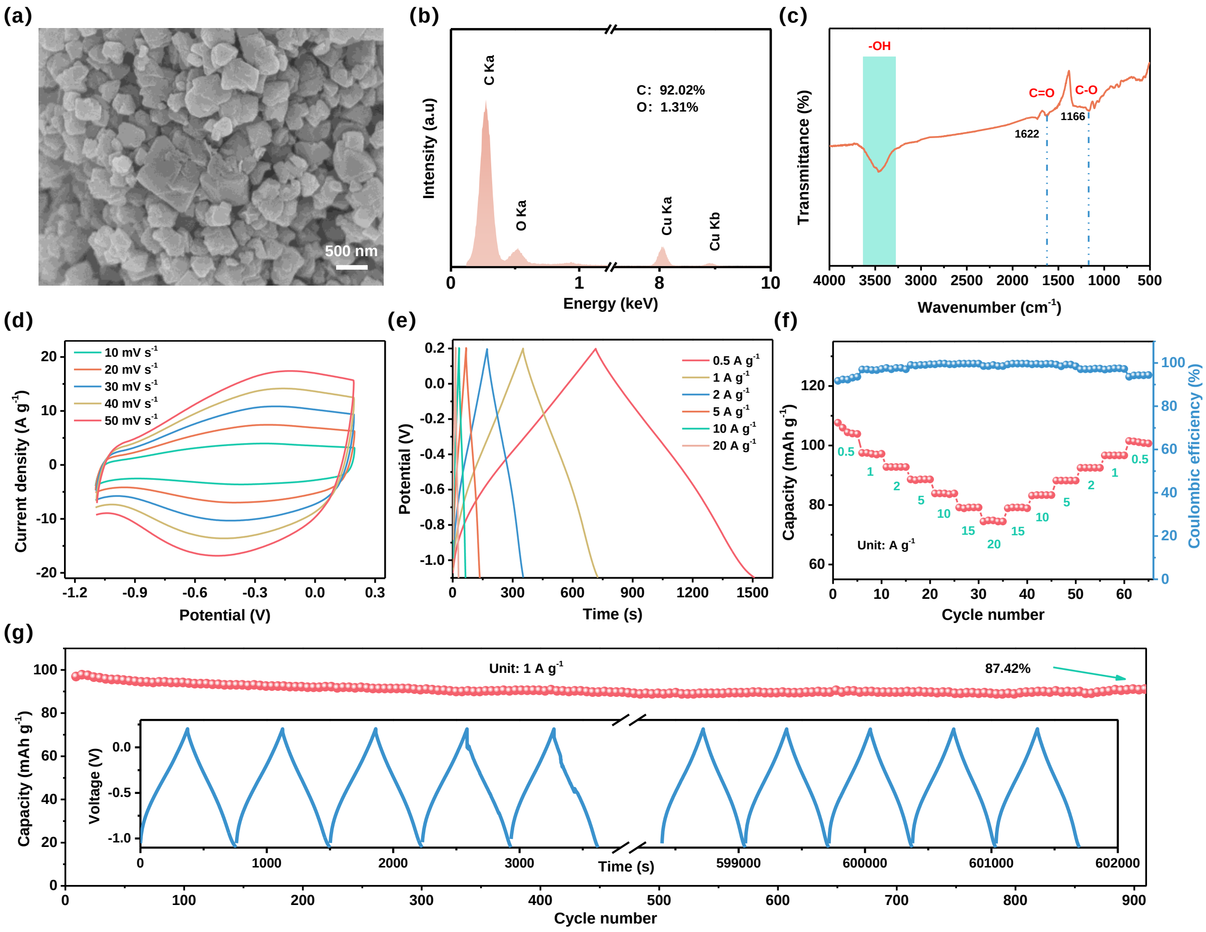

Figure S29. a) SEM image, b) EDS and c) FT-IR spectrum of ZTC anode. d) The CV curves of activated ZTC anode at different scan rates. e) The GCD curves of activated ZTC anode at different current densities. f) Rate capability and g) cycling performance at 1 A g^{-1} of activated ZTC anode.

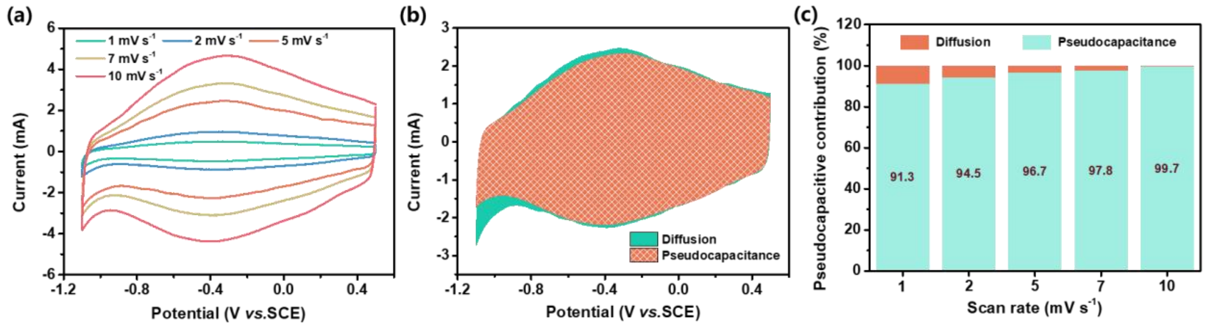

Figure S30. a) The CV curves of activated ZTC anode at different scan rates within potential window of -1.1~0.4 V. b) Capacitive (orange) and diffusion-controlled (green) contributions to charge storage at 5 mV s^{-1}. c) Capacitive contribution percentage at various scan rates.

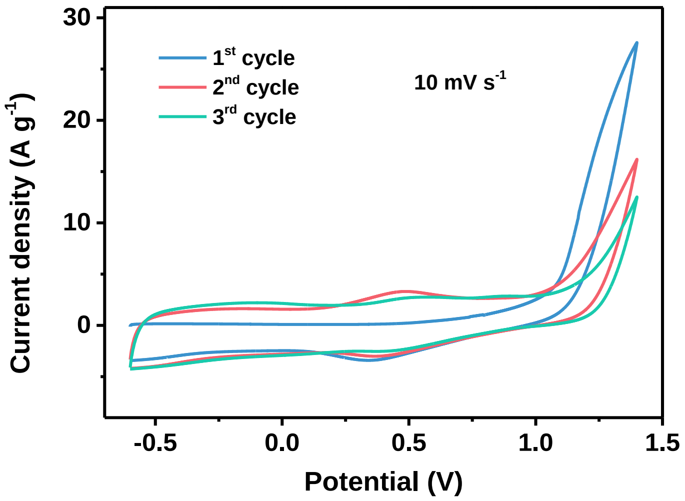

Figure S31. The activation process of ZTC anode via CV cycling.

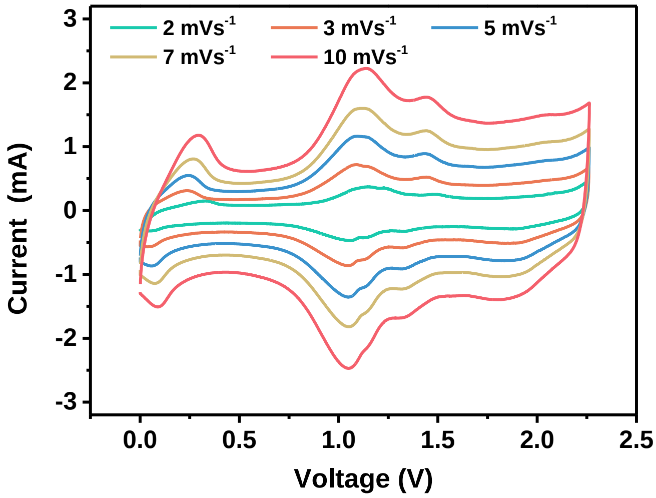

Figure S32. CV profiles of ALD-AL//ZTC ASIHC at different scan rates from 2 to 10 mV s^{-1}.

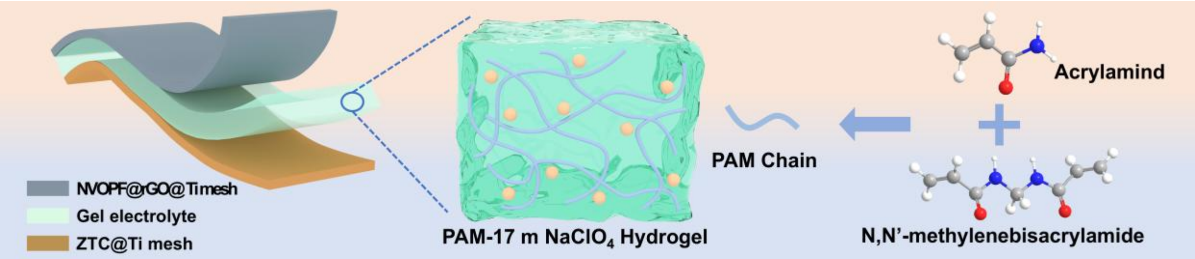

Figure S33. Schematic illustration of the QASIHC device with PAM-17 m $NaClO_4$ hydrogel electrolyte.

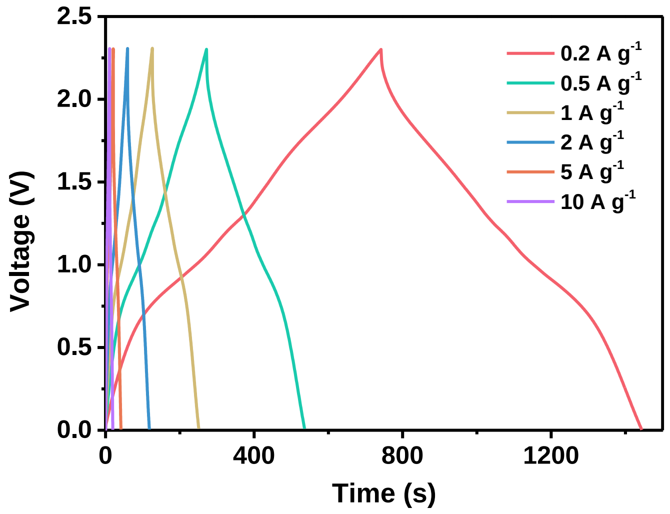

Figure S34. GCD curves of QASIHC at various current densities from 0.2 to 10 A g^{-1}.

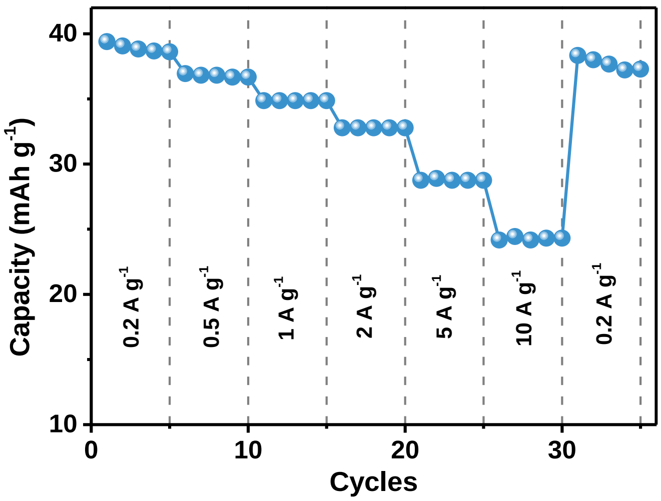

Figure S35. Rate performance of our QASIHC device.

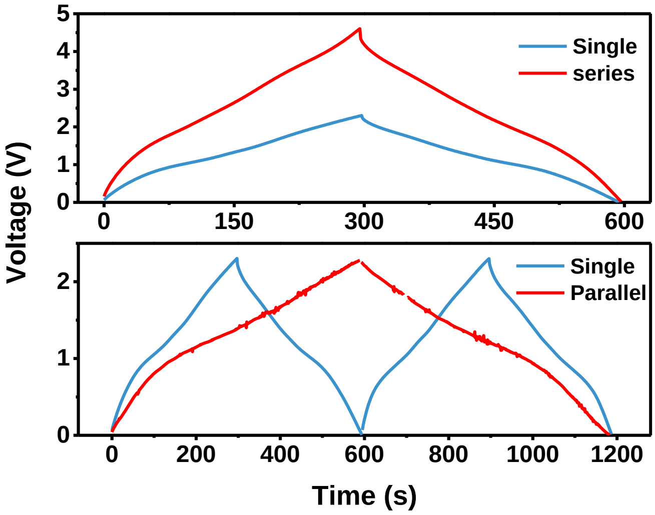

Figure S36. The corresponding GCD curves of QASIHC pouch cells combined in series or in parallel.

Table S1. Rietveld refinement parameters of pristine and ALD-AL cathodes.

Sample	*a* (Å)	*c* (Å)	R_{wp} (%)	R_p (%)
**Pristine**	9.044	10.653	11.8	5.56
**ALD-AL**	9.045	10.654	7.85	4.1

Table S2. The performance comparison of various NVOPF-based cathodes.

Cathode materials	Electrolyte	Cut-off voltage (V)	Rate (C)	Maximum capacity (mA h g^{-1})	Cycle performance	Coulombic efficiency (%)
NVPF-SWCNT (Shuang, L)	10 M $NaClO_4$ +0.25% CMC	1.2	1	81.3	60, 71.3%	84.2
NVOPF-MWCNT (Kumar, P. R)	1 M Na_2SO_4	0.9	1	48	100, 35.4%	86.5
NVOPF@rGO (Gong, P)	17 m $NaClO_4$	1.0	1	46.4	100, 92.9%	82.3
**NVOPF@rGO (Pristine)**	**17 m $NaClO_4$**	**1.2**	**1**	**83.9**	**40, 23.3%**	**60.1**
**Al_2O_3-NVOPF@rGO (ALD-AL)**	**17 m $NaClO_4$**	**1.2**	**1**	**91.9**	**100, 54.9%**	**92.3**

Table S3. Impedance parameters of pristine and ALD-AL cathodes after three cycles obtained from equivalent circuit fittings.

Sample	R_s (Ω)	R_{CEI} (Ω)	R_{ct} (Ω)
**Pristine**	0.897	0.759	7.613
**ALD-AL**	0.913	0.465	1.959

Table S4. Impedance parameters of ALD-AL cathode after different cycles obtained from equivalent circuit fittings.

Sample	R_s (Ω)	R_{CEI} (Ω)	R_{ct} (Ω)
**After 1 cycle**	0.658	0.648	3.831
**After 10 cycles**	0.685	0.644	3.850
**After 30 cycles**	0.699	0.639	3.879
**After 50 cycles**	0.713	0.636	3.894
**After 100 cycles**	0.699	0.625	3.911

Table S5. Electrochemical performance comparison of the reported ASIHCs.

**Cathode//Anode[Ref.]**	**Electrolyte**	**Voltage (V)**	**Capacity at maximum current density**
Na_XMnO_2-CNT//AC [1]	1 M Na_2SO_4	1.8	9.4 mA h g^{-1} at 5 A g^{-1}
$Na_{2/3}MnO_2$//AC [2]	1 M Na_2SO_4	2	8.9 mA h g^{-1} at 4 A g^{-1}
AC//CF@VN [3]	2 M KOH	1.6	6.67 mA h g^{-1} at 5 A g^{-1}
$NiCo_2O_4$//AC [4]	2 M KOH	1.4	10.89 mA h g^{-1} at 4 A g^{-1}
ATA//PDI-Ph [5]	30 m NH_4AC	1.9	12 mA h g^{-1} at 2 A g^{-1}
NVO//AC [6]	2 M $ZnSO_4$	2	20.3 mA h g^{-1} at 2 A g^{-1}
ZnS-$CuSe_2$//AC [7]	3 M KOH	1.7	16.5 mA h g^{-1} at 5 A g^{-1}
$ZnCr_2O_4$//AC [8]	1 M KOH	1.6	13.1 mA h g^{-1} at 10 A g^{-1}
$NaMnO_2$//$NaTi_2(PO_4)_3$ [9]	2 M NaAC	1.8	20 mA h g^{-1} at 1.2 A g^{-1}
**This work (ALD-AL//ZTC)**	**17 m $NaClO_4$**	**2.3**	**23.75 mA h g^{-1} at 10 A g^{-1}**

References:

[1] J. A. Wang, C. C. Ma, C. C. Hu, *Electrochim. Acta* **2020**, 334, 135576.
[2] A. A. Nechikott, P. K. Nayak, *RSC Adv.* **2023**, 13, 14139.
[3] Y. Wang, M. Jiang, Y. Yang, F. Ran, *Electrochim Acta* **2016**, 222, 1914.
[4] R. Ding, L. Qi, M. Jia, H. Wang, *Electrochim Acta* **2013**, 107, 494.
[5] K. S. Lakshmi, X. Ji, T. Y. Chen, B. Vedhanarayanan, T. W. Lin, *J. Power Sources* **2021**, 511, 230434.
[6] X. Liang, J. Li, X. Yang, L. Wang, X. Li, W. Lu, *J. Energy Storage* **2022**, 56, 105947.
[7] S. A. Ahmad, M. U. Shah, M. Arif, E. Ullah, S. Rahman, M. U. Shah, S. M. Eldin, P. Song, M. Sajjad, A. Shah, *Ceram. Int.* **2023**, 49, 20007.
[8] T. Fei, T. Ahmad, M. Usman, A. Ahmad, A. Saleem, M. B. Hanif, A. M. Karami, M. S. Javed, B. Akkinepally, C. Xia, *Electrochim. Acta* **2024**, 476, 143673.
[9] Z. Hou, X. Li, J. Liang, Y. Zhu, Y. Qian, *J. Mater. Chem. A* **2015**, 3, 1400.
